# Norepinephrine transporter-derived homing peptides enable rapid endocytosis of drug delivery nanovehicles into neuroblastoma cells

**DOI:** 10.1186/s12951-020-00654-x

**Published:** 2020-07-13

**Authors:** Yazan Haddad, Marketa Charousova, Hana Zivotska, Zbynek Splichal, Miguel Angel Merlos Rodrigo, Hana Michalkova, Sona Krizkova, Barbora Tesarova, Lukas Richtera, Petr Vitek, Kamila Stokowa-Soltys, David Hynek, Vedran Milosavljevic, Simona Rex, Zbynek Heger

**Affiliations:** 1 Department of Chemistry and Biochemistry, Mendel University in Brno, Zemedelska 1, 613 00 Brno, Czechia; 2 Central European Institute of Technology, Brno University of Technology, Purkynova 123, 612 00 Brno, Czechia; 3 Global Change Research Institute of the Czech Academy of Sciences, Belidla 986/4a, 603 00 Brno, Czechia; 4 Faculty of Chemistry, University of Wrocław, F. Joliot-Curie 14, 50-383 Wrocław, Poland

**Keywords:** Homing peptide, Ferritin, Neuroblastoma, Norepinephrine transporter, Targeted therapy

## Abstract

**Background:**

Currently, the diagnosis and treatment of neuroblastomas—the most frequent solid tumors in children—exploit the norepinephrine transporter (hNET) via radiolabeled norepinephrine analogs. We aim to develop a nanomedicine-based strategy towards precision therapy by targeting hNET cell-surface protein with hNET-derived homing peptides.

**Results:**

The peptides (*seq.* GASNGINAYL and SLWERLAYGI) were shown to bind high-resolution homology models of hNET in silico. In particular, one unique binding site has marked the sequence and structural similarities of both peptides, while most of the contribution to the interaction was attributed to the electrostatic energy of Asn and Arg (< − 228 kJ/mol). The peptides were comprehensively characterized by computational and spectroscopic methods showing ~ 21% β-sheets/aggregation for GASNGINAYL and ~ 27% α-helix for SLWERLAYGI. After decorating 12-nm ferritin-based nanovehicles with cysteinated peptides, both peptides exhibited high potential for use in actively targeted neuroblastoma nanotherapy with exceptional in vitro biocompatibility and stability, showing minor yet distinct influences of the peptides on the global expression profiles. Upon binding to hNET with fast binding kinetics, GASNGINAYLC peptides enabled rapid endocytosis of ferritins into neuroblastoma cells, leading to apoptosis due to increased selective cytotoxicity of transported payload ellipticine. Peptide-coated nanovehicles significantly showed higher levels of early apoptosis after 6 h than non-coated nanovehicles (11% and 7.3%, respectively). Furthermore, targeting with the GASNGINAYLC peptide led to significantly higher degree of late apoptosis compared to the SLWERLAYGIC peptide (9.3% and 4.4%, respectively). These findings were supported by increased formation of reactive oxygen species, down-regulation of survivin and Bcl-2 and up-regulated p53.

**Conclusion:**

This novel homing nanovehicle employing GASNGINAYLC peptide was shown to induce rapid endocytosis of ellipticine-loaded ferritins into neuroblastoma cells in selective fashion and with successful payload. Future homing peptide development via lead optimization and functional analysis can pave the way towards efficient peptide-based active delivery of nanomedicines to neuroblastoma cells.

## Background

Neuroblastomas, originating in the sympathetic nervous system, belong to the most frequent solid tumors among children under the age of five with a survival median of 23 months [1]. Neuroblastomas belong to the peripheral neuroblastic tumors (PNTs) [2]. While most of the members of the PNTs family are benign and curable by surgery, neuroblastomas and uncommon nodular ganglioneuroblastomas are the exceptions to this [3].

The most common location of neuroblastoma is in the adrenal medulla and along sympathetic ganglia. Patient’s symptoms can indicate tumor location, with masses most often in pelvis, retroperitoneum, thorax, neck or bones; causing urinal retention, constipation, changes in activity and movements [3]. Neuroblastoma treatment is based on a combination of surgery, chemotherapy, radiotherapy and biotherapy. Surgery is only viable for localized neuroblastoma and can lead to complications or even death [4]. Biotherapy employs antibodies against certain targets [5], however, concerns about the immunogenicity are being raised where antibodies are used [6]. Conventional chemotherapy leads to various adverse side effects due to non-specificity of the drug’s mechanism of action. One of the approaches to combat this phenomenon is the employment of drug delivery systems, targeting specific receptors overexpressed on the cancer cells surface [7]. Another approach is the application of radiolabeled drugs, such as metaiodobenzylguanidine (MIBG), one of the most commonly utilized theranostic modality for neuroblastomas. Two iodine isotopes of MIBG, namely ^123^I-MIBG and ^131^I-MIBG, are used for diagnostic imaging and therapy, respectively [8]. ^131^I-MIBG is the major course of treatment for metastasized and high risk neuroblastomas, which at the time of clinical presentation constitutes nearly half the neuroblastoma patients. Even then, this form of radiotherapy leads to dose-related side effects, due to influencing bone marrow production, hepatotoxicity and risk of secondary malignancies. MIBG is a guanethidine analog of norepinephrine, targeting the human norepinephrine transporter (hNET), a transmembrane protein encoded by the *SLC6A2* gene. hNET is responsible for the reuptake and clearance of norepinephrine [9] and is expressed by several malignancies of neuroendocrine origin, including neuroblastomas [10] with estimates of nearly 90% expression among neuroblastoma cells [11]. hNET and other monoamine transporters were shown to form homo-oligomers and cluster in specialized areas of the plasma membrane [12]. This phenomenon was the motivation behind using hNET fragments to design the presented homing peptides. We hypothesized that hNET-derived fragments may possess complementary sequences or self-interacting domains that play role in either protein folding or homo-oligomerization. Furthermore, due to the lack of known crystal structure of hNET, a homology modeling approach was required to construct a 3-D model using crystal structure templates of the closest known homologs, i.e., drosophila dopamine transporter (dDAT) [13] and human serotonin transporter (hSERT) [14].

Ellipticine (Elli) is considered one of the best candidate payload drug for use in drug delivery nanocarriers. It is known for its high antitumor activity, yet Elli has had limited clinical application in the past due to its non-specific nature leading to toxic adverse effects. Nevertheless, drug delivery systems can safeguard against off-target toxicity [15].

In order to develop a nanomedicine-based strategy towards precision therapy of neuroblastoma, we have targeted hNET cell-surface protein by constructing a ferritin (FRT)-based nanovehicle carrying a payload of Elli. Homing peptides were connected to the surface of nanovehicle via gold nanoparticles (AuNPs). The design of hNET-homing peptides from hNET fragments, as mentioned earlier, was done using homology modeling and molecular docking. The peptide sequence is spanning residues 286–295 (GASNGINAYL) and 583–592 (SLWERLAYGI) of hNET protein (Uniprot ID: P23975). Peptides were characterized by molecular dynamics (MD) and by various spectroscopic methods. Cytotoxicity and biocompatibility were also evaluated, showing minor yet distinct influences of the peptides on the global expression profiles. The selective delivery of a non-specifically cytotoxic Elli into neuroblastoma cells was performed via peptide-decorated 12-nm FRT-based nanovehicle. The hNET-homing peptides enabled rapid uptake of the nanovehicles into neuroblastoma cells with induced hNET expression through endocytosis. Most importantly, the peptides markedly increased the cytotoxicity of the encapsulated Elli for neuroblastoma cells, while simultaneously enhancing its biocompatibility. Overall, the obtained data underpin that the developed peptides are highly promising targeting ligands for use in neuroblastoma nanomedicine.

## Results

### Homing peptides bind to hNET in silico

**Fig. 1:**
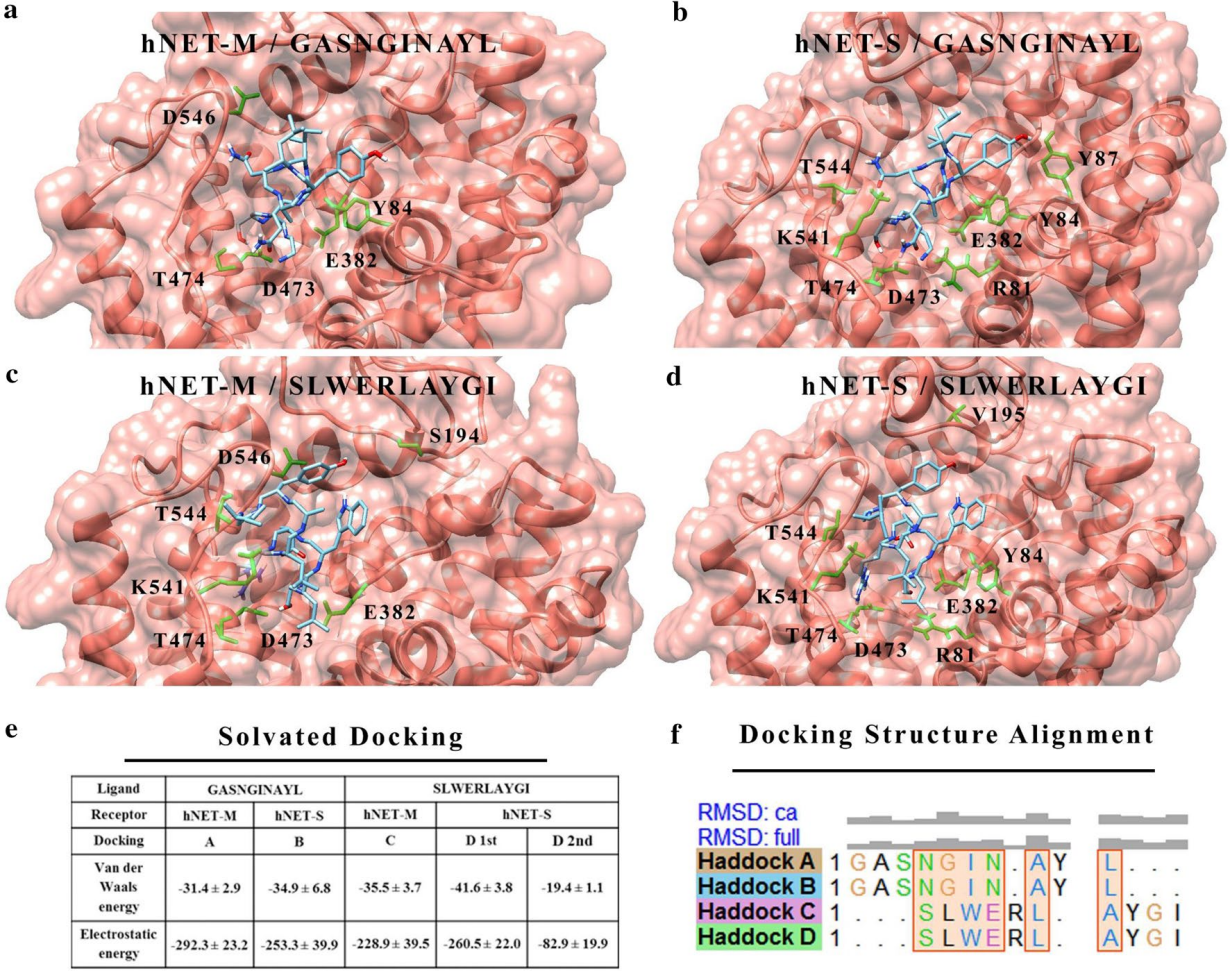
Haddock solvated docking shows binding of hNET-derived peptides to hNET in silico. Best-predicted binding pose of GASNGINAYL peptide with hNET-M model (**a**) and hNET-S model (**b**), and for SLWERLAYGI peptide with hNET-M model (**c**) and hNET-S model (**d**). Peptides are shown with minimal backbone for clarity (light blue—backbone, red—oxygen, dark blue—nitrogen). hNET ribbon and surface are shown in salmon-pink. hNET side-chains within 3 Å distance from peptides are labeled and shown in green. **e** Non-bonded energies showing major contribution of electrostatic interactions to the binding. **f** Structure–Structure alignment of top four binding poses indicating similar mode of interaction. The overlapping sequence between the homing peptides is attributed to six homologous residues (highlighted boxes) at the same binding site. Root-mean square-deviations (RMSD) are shown in figurative grey bars for backbone (Ca) and for both backbone and side-chains (Full). The alignment can serve as a roadmap for designing derived sequences of hNET homing peptides

Using dDAT 3-D structure as a template, we built and refined two alternative homology models of hNET: (i) hNET-M (constructed using Modeller) [16] and (ii) hNET-S (constructed using SwissModel) [17]. For both models, the sequence-structure alignment in Modeller was used to thread the hNET sequence to the backbone of dDAT structure (Additional file 1: Figure S1). hNET-M and hNET-S were manually refined according to previously published strategy [18]. This strategy incorporates additional structural information from the homologous hSERT crystal structure without jeopardizing the MolProbity [19] quality score. Final MolProbity scores, normalized to reflect X-ray resolution, were 2.15 Å and 1.6 Å for hNET-M and hNET-S homology models, respectively (Additional file 1: Table S1). Preliminary ClustPro [20] docking of homing peptides with hNET homology models showed binding clusters inside the channel of hNET model in more than 20% of 1000 trials for both peptides and both homology models (Additional file 1: Table S2). These dockings were used to predict a site-specific binding via Haddock [21] solvated docking (Fig. 1 and Additional file 1: Table S3). Haddock docking was guided to use the peptides’ *N*-termini to target D473 (by GASNGINAYL) and E382 (by SLWERLAYGI) residues in hNET (Fig. 1a–d). Results revealed a major contribution of electrostatic energy to the binding compared to Van der Waals energy (Fig. 1e and Additional file 1: Table S3). The highest contributions were clearly from positively charged Asn residues in GASNGINAYL and Arg in SLWERLAYGI. Structure-structure alignment highlighted the homology in sequence and structure between both peptides indicating a similar mode of interaction with hNET (Fig. 1f). Both peptides were helical in the best-predicted binding poses.

### GASNGINAYL peptide aggregates while SLWERLAYGIC peptide forms a stable α-helix

In order to utilize the homing peptides, two chemical modifications were required. Firstly, the addition of amide group at the *C*-terminus was required to protect peptides from proteases in biological environment. Secondly, the addition of Cys residue at the *C*-terminus was required to facilitate the linking of peptides to AuNPs. The consequences of these modifications on peptide secondary structure were investigated computationally by molecular dynamics and physically by spectroscopic methods.

**Fig. 2:**
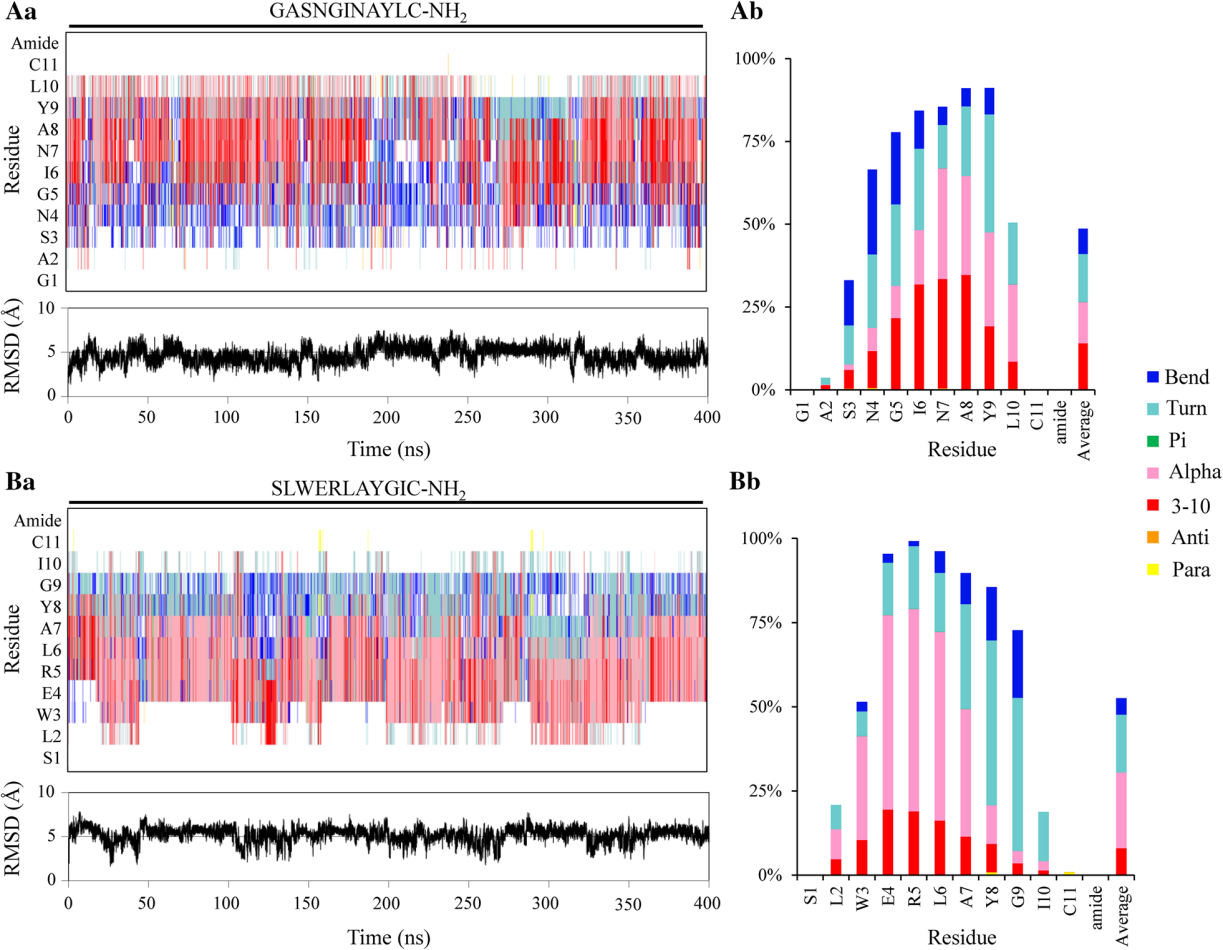
Nearly 50% of secondary structure assignments were random coils, while the rest were attributed to α-helix, 3-10 helix, turn and bend. Generalized Born implicit-water MD simulations (400 ns) at physiological pH 7.4 of cysteinated and *C*-terminal amidated homing peptides GASNGINAYLC-NH_2_ (**A**, **a**–**b**) and SLWERLAYGIC-NH_2_ (**B**, **a**–**b**). **a** Time evolution of peptide secondary structures and root-mean square-deviations (RMSD, Å). **b** Percentage of secondary structure distribution among residues showing assignments of known secondary structures in nearly 50% of the simulation time (Average in the last column). The first and last residues have neither backbone dihedrals nor assignments, and were calculated as zero in the average for uniformity

MD simulations of peptides demonstrated helices, bends and turns in nearly 50% of the time (Fig. 2). The remaining 50% were not assigned to any secondary structure (Fig. 2Ab, Bb), which indicates the flexibility of these peptides (particularly at the terminal residues) in implicit water simulations. All-atom RMSD deviation values from the standard α-helix reference were observed vacillating around the 5 Å RMSD (e.g. Figure 2, *bottom left panels*). Relatively, cysteination improved the helicity of peptides in simulations (Fig. 2 and Additional file 1: Figure S2). For GASNGINAYL(C) variants, α-helix assignments increased from 2.5% in control to 7.3%, 8.0% or 12.5% by addition of Cys, amide, or both, respectively (Fig. 2 and Additional file 1: Figure S2). The 3–10 helix assignments changed from 17.4% in control to 18.1%, 17.0% or 13.8% by addition of Cys, amide, or both, respectively (Fig. 2 and Additional file 1: Figure S2). For SLWERLAYGI(C) variants, α-helix assignments varied from 21.0% in control to 34.8%, 20.8% or 22.5% by addition of Cys, amide, or both, respectively (Fig. 2 and Additional file 1: Figure S2). The 3–10 helix assignments changed from 9.5% in control to 4.9%, 9.2% or 7.9% by addition of Cys, amide, or both, respectively (Fig. 2 and Additional file 1: Figure S2).

**Fig. 3:**
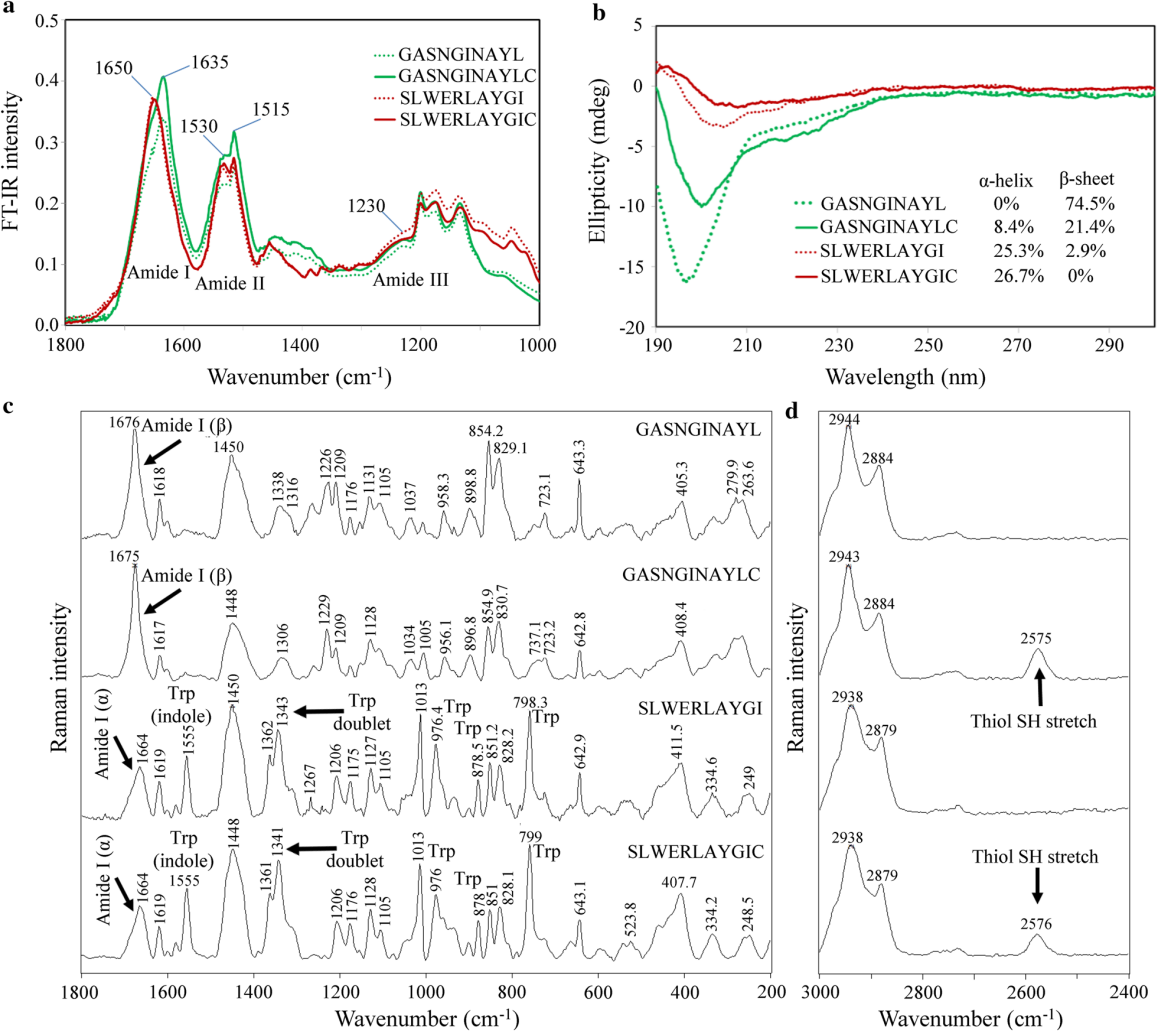
Spectral characterization of homing peptides shows aggregation for GASNGINAYL(C) and α-helices for SLWERLAYGI(C). **a** FT-IR spectrum. **b** CD spectrum. **c** Raman spectrum in the range of 1800–200 cm^−1^. **d** Raman spectrum in the range of 3000–2400 cm^−1^

As shown by the experimental secondary structure assignment using Fourier transform-infrared spectroscopy (FT-IR), the amide I (wavenumber range of 1490–1590 cm^−1^) and amide II (wavenumber range of 1590–1700 cm^−1^) bands were easily identified (Fig. 3a). The wide base of both bands was in accordance with the foreseen dynamic peptide structures. Both SLWERLAYGI(C) variants showed highly similar amide I band that strongly correlates with α-helix structure region around 1650 cm^−1^ (Fig. 3a), and indicates no significant secondary structural change as a result of adding Cys. Similarity between the amide I spectrum of GASNGINAYL(C) variants indicates no significant secondary structural shift because of adding Cys. However, clues of α-helix structure appear as a mild shoulder to a more predominant peak in the β-sheet/backbone-aggregation region around 1630–1640 cm^−1^ (Fig. 3a).

Circular Dichroism (CD) spectra showed positive cotton effect at 192 nm in the SLWERLAYGI(C) variants, while negative cotton effect at 207 and 222 nm was observed in all peptide variants (Fig. 3b). Addition of Cys improved the helicity in all peptide variants. GASNGINAYLC and SLWERLAYGIC displayed 8.4% and 26.7% α-helix, respectively (Fig. 3b). Interestingly, GASNGINAYLC showed ~ 53% decrease in contribution of β-sheets due to addition of Cys residue, indicating that Cys disrupted inter- or intra-chain interactions.

Raman spectra displayed distinct amide and side-chain vibrations for each of the studied peptides (Fig. 3c). Secondary structure assignments confirmed previous findings of β-sheet signatures in GASNGINAYL(C) variants at 1676–1675 cm^−1^. Furthermore, a clear band at 1664 cm^−1^ corresponded to α-helix in SLWERLAYGI(C) variants. Predominant bands of Trp in the SLWERLAYGI(C) variants were also observed at 1555, 1343–1341, 976 and 759–758 cm^−1^ (Fig. 3c). A free thiol (-SH) stretch vibration was assigned to the GASNGINAYLC (at 2575 cm^−1^) and SLWERLAYGIC (at 2576 cm^−1^) peptides (Fig. 3d).

### Homing peptides show negligible cytotoxicity but distinctly influence the global expression profiles in neuroblastoma

**Fig. 4:**
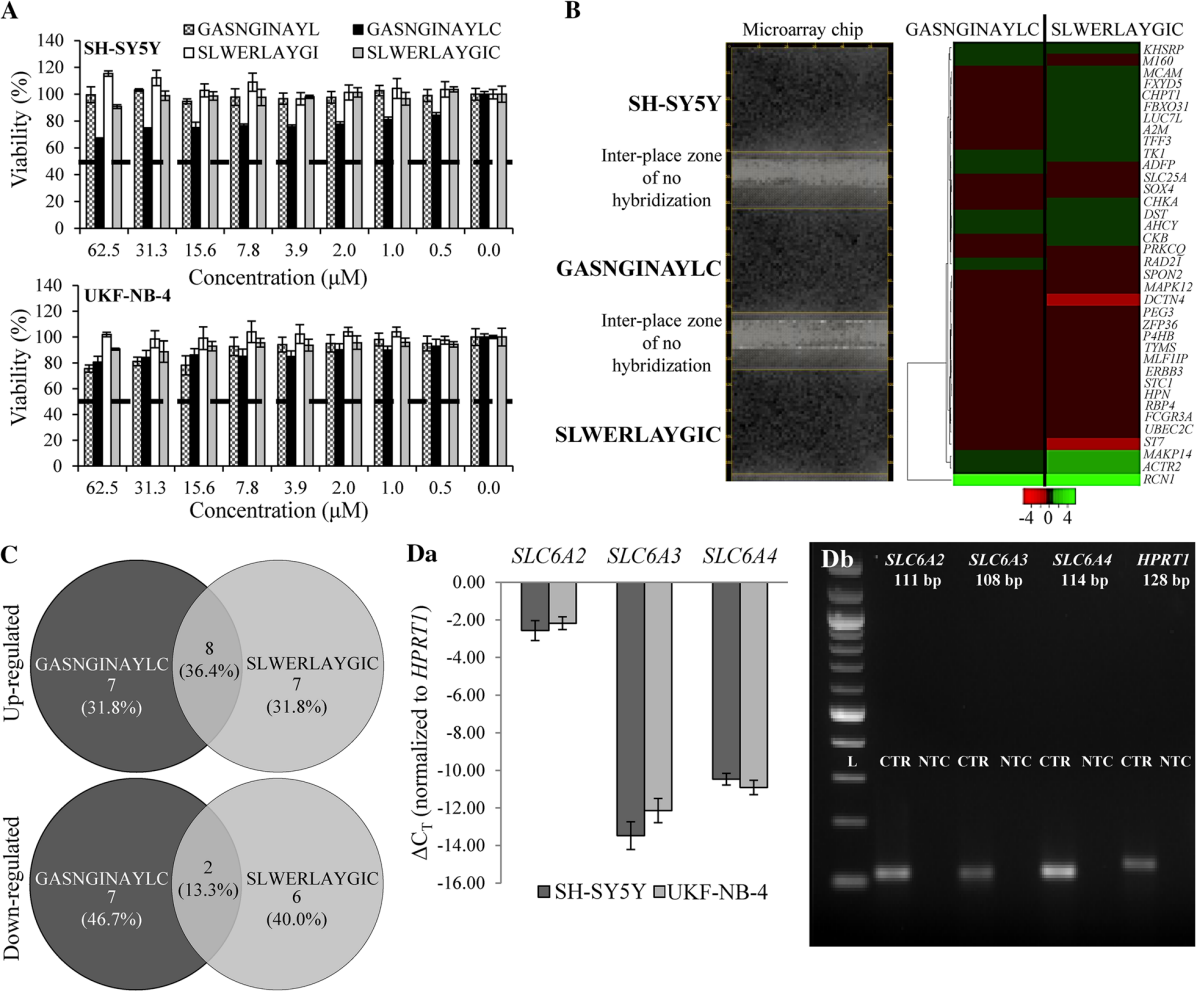
hNET-homing peptides do not display a direct cytotoxicity in neuroblastoma cells. **a** Cytotoxicity profiles of varying concentrations of peptides GASNGINAYL, GASNGINAYLC, SLWERLAYGI or SLWERLAYGIC for SH-SY5Y (*upper panel*) and UKF-NB-4 (*lower panel*) cells upon 24 h treatment. **b** Representative microarray (*left panel*) and expression heatmap (*right panel*) revealing expression of cancer-biomarker genes (one spot per one gene) in non-treated SH-SY5Y cells, and SH-SY5Y cells treated with GASNGINAYLC and SLWERLAYGIC peptides. **c** Venn diagram summarizing the number of up- (**Ca**) and down-regulated (**Cb**) genes by both or individual hNET-homing peptides. (**Da**) qPCR showing differences in relative expression of monoamine transporter genes *SLC6A2* (encoding hNET), *SLC6A3* (encoding hDAT) and *SLC6A4* (encoding hSERT) in neuroblastoma cell lines (expression normalized to *HPRT1*) (**Db**) Electrophoretic analysis of qPCR amplicons. L—100 bp DNA molecular weight marker. CTR—qPCR amplicons from UKF-NB-4 cDNA. *NTC* non-template control

The in vitro cytotoxicity testing of the synthesized homing peptides (GASNGINAYL, GASNGINAYLC, SLWERLAYGI or SLWERLAYGIC) for neuroblastoma cells UKF-NB-4 and SH-SY5Y showed cytotoxicity after 24 h treatment only for high concentrations of the peptides (Fig. 4a). Furthermore, only small differences were observed between the cytotoxicity of the cysteinated and non-cysteinated SLWERLAYGI peptide. Therefore, due to their ability to decorate FRT surface in a site-directed manner, only cysteinated peptides were tested in the follow-up experiments. The concentrations used for this decoration were in the range of very low cytotoxicity.

We further investigated the effect of GASNGINAYLC and SLWERLAYGIC peptides on cancer-related transcriptome by parallel cDNA microarray profiling (representative microarray heatmaps are shown in Fig. 4b). Venn diagrams were constructed from complete lists of genes differentially expressed due to hNET-homing peptides administration (complete lists are shown in Additional file 1: Table S4 and Additional file 1: Table S5), and indicated only a partially shared response of SH-SY5Y cells to the treatment (8 up- and 2 down-regulated common genes, Fig. 4c). To simplify the insight into the effect of the hNET-homing peptides on SH-SY5Y cells, we further analyzed the biological processes and pathways affected by differentially expressed genes. Table 1 shows that the peptides stimulated the expression of genes involved in metabolic processes, response to stress and nervous system development, whereas the down-regulated genes were mostly connected with intracellular signaling, cellular response to stimulus and cellular senescence.

**Table 1:** Classification of cellular pathways affected by differentially expressed genes identified in SH-SY5Y cells as predicted by DAVID software

Gene ontology ID	Cellular pathway	Genes	FDR
GASNGINAYLC			
Up-regulated genes			
GO.0008152	Metabolic process	7	0.0014
GO.0006950	Response to stress	7	0.0001
GO.0009605	Response to external stimulus	3	0.0006
GO.0007399	Nervous system development	3	0.0081
Down-regulated genes			
GO.0006508	Proteolysis	3	0.0307
GO.0030198	Extracellular matrix organization	2	0.0027
GO.0007165	Diseases of signal transduction	2	0.0081
GO.0090398	Cellular senescence	1	0.0003
SLWERLAYGIC			
Up-regulated genes			
GO.0044707	Single-multicellular organism process	10	0.0015
GO.0006950	Response to stress	8	0.0004
GO.0009653	Anatomical structure morphogenesis	4	0.0105
GO.0007399	Nervous system development	3	0.0430
GO.0048699	Generation of neurons	2	0.0266
Down-regulated genes			
GO.0051716	Cellular response to stimulus	6	0.0319
GO.0035556	Intracellular signal transduction	3	0.0024
GO.0007264	Small GTPase mediated signal transduction	2	0.0005

*FDR* false discovery rate

In addition, expression profiling of hNET, hDAT and hSERT revealed similar expression level of hNET in both tested cell lines (Fig. 4Da, product control is demonstrated in Fig. 4Db). Furthermore, we revealed a marked overexpression of hNET compared with both hDAT (> 900×) and hSERT (> 230×). This proved hNET as a selective target in both neuroblastoma cell lines without expected contributions from hSERT and hDAT which were used to construct the high-resolution homology models.

### FRT-based nanovehicles can bind cysteinated hNET-homing peptides

**Fig. 5:**
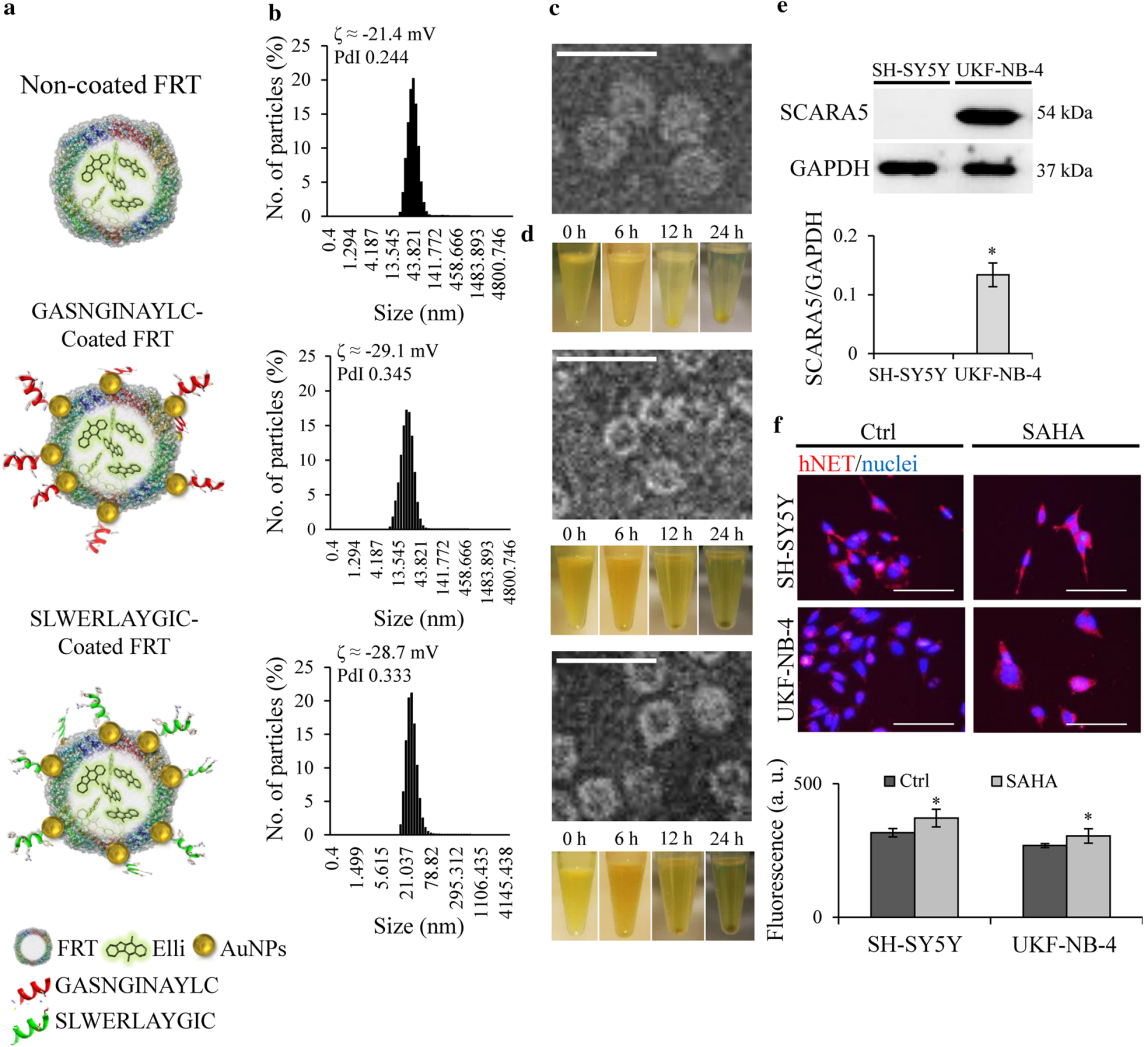
Cysteinated hNET-homing peptides can bind to AuNPs-decorated FRT-nanovehicles in site-directed orientation. **a** Schematic representation of naked FRT with encapsulated Elli (FRT-Elli, upper), FRT-Elli modified with GASNGINAYLC (*middle*) and FRT-Elli modified with SLWERLAYGIC (*lower*). **b** Size, polydispersity index and ζ-potential. **c** Transmission electron micrographs. Scale bar, 25 nm. **d** Colloidal stability for up to 24 h in Ringer’s solution at 20 °C. **e** Relative expression of SCARA5 in neuroblastoma cell lines. *indicates significant (*p *< 0.005) difference between SH-SY5Y and UKF-NB-4 cells. **f** Immunofluorescence of hNET (red) in control neuroblastoma cells and the same cells after 24 h treatment with 10 µM suberanilohydroxamic acid (SAHA). Hoechst 33342 was used to counterstain nuclei (blue). Scale bar, 50 µm. *indicates significantly (*p *< 0.05) increased hNET expression after SAHA treatment

To evaluate the hNET-homing properties of the GASNGINAYLC and SLWERLAYGIC peptides, they were attached via cysteine in a site-directed orientation to a horse spleen FRT nanovehicles decorated with adsorbed 1.3 nm AuNPs. This hNET-homing nanovehicle was used to deliver highly cytotoxic compound Elli (with encapsulation efficiency of approximately 55% for all prepared nanovehicles, resulting in 720 µM Elli per 3.5 µM FRT). Figure 5a shows schematic representation of the prepared nanovehicles.

The binding of hNET-homing peptides onto FRT nanovehicles resulted in significant improvement of surface ζ-potential towards higher stability of the nanovehicles (Fig. 5b). This also led to increased repulsion among FRT-Elli nanovehicles and formation of smaller vehicles, from ~ 43 nm for FRT-Elli to ~ 21 nm and ~ 28 nm for FRT-Elli-GASNGINAYLC and FRT-Elli-SLWERLAYGIC, respectively. The changes in ζ-potential upon addition of peptides correlated with predicted pI of peptides (pI = 5.52 for GASNGINAYLC and 5.72 for SLWERLAYGIC, not adjusted for loss of basic thiol group) confirming the increased negative charge on the nanocarrier surface.

Furthermore, the overall icosahedral shape of FRT cage was not changed by either Elli encapsulation, or the introduction of hNET-homing peptides (Fig. 5c). Also, due to the increase in the surface ζ-potential, the colloidal stability of these hNET-homing nanovehicles was prolonged and no precipitation or aggregate formation was observed even after 24 h in plasma-simulating Ringer’s solution, only sedimentation of heavy Elli-encapsulating FRT (Fig. 5d).

Next, the expression patterns of surface receptors were evaluated to choose a suitable neuroblastoma cell line for further experiments. The cells varied significantly in the expression of scavenger receptor protein SCARA5, a predominant receptor for the *L*-subunit-rich horse spleen FRT (Fig. 5e), with a very high expression in UKF-NB-4. On the other hand, SH-SY5Y cells showed higher level of hNET expression, which increased even further after treatment with inducer of hNET expression suberanilohydroxamic acid (SAHA) (Fig. 5f, control micrographs are shown in Additional file 1: Figure S3). Taken together, results showed a better suitability of SH-SY5Y cells for further hNET-homing studies.

### hNET-homing peptides enable rapid endocytosis of nanovehicles into neuroblastoma cells

**Fig. 6:**
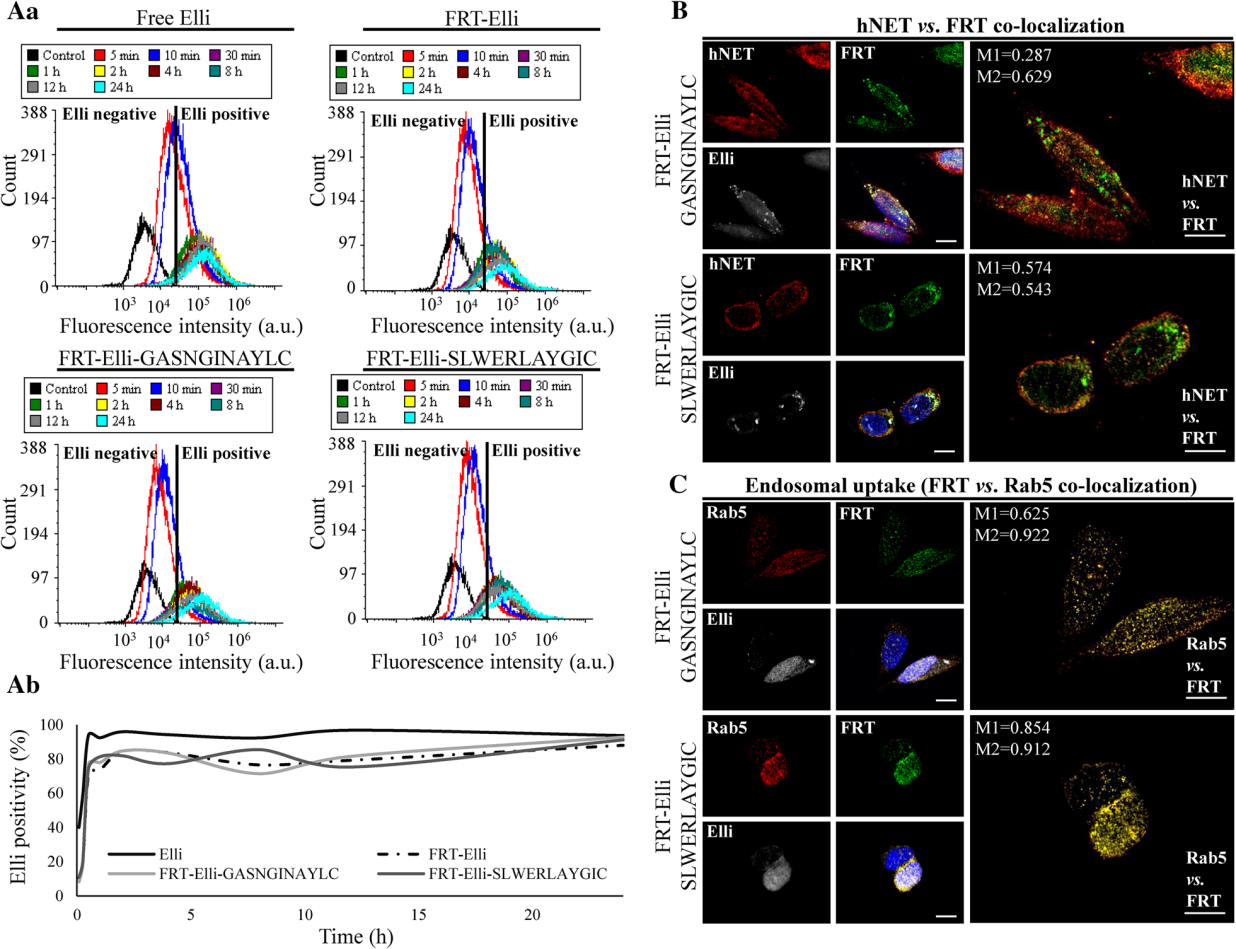
hNET-homing nanovehicles are able to induce endocytosis of hNET in SH-SY5Y cells. **a** Uptake kinetics of 20 µM Elli, FRT-Elli, FRT-Elli-GASNGINAYLC and FRT-Elli-SLWERLAYGIC observed over 24 h revealed by flow cytometry. **Aa** Elli fluorescence intensity at various time points. **Ab** The percentage of Elli-positive cells. **b** Intracellular trafficking of FRT (green) stained with Cy3 NHS ester and encapsulating Elli (80 µM, white), immunofluorescence of hNET (red) and co-localization of FRT and hNET. Hoechst 33342 was used to counterstain nuclei (blue). Scale bar, 10 µm. Manders’ coefficient M1 shows overlapping of hNET on FRT. Manders’ coefficient M2 shows overlapping of FRT on hNET. **c** Co-localization of FRT (green) encapsulating Elli (white) and immunofluorescence of early endosomal marker Rab5 (red). Hoechst 33342 was used to counterstain nuclei (blue). Scale bar 10 µm. Manders’ coefficient M1 shows overlapping of Rab5 on FRT. Manders’ coefficient M2 shows overlapping of FRT on Rab5. Manders’ coefficients were taken for the representative images only

The association of hNET-homing nanovehicles with neuroblastoma cells in presence of serum was rapid, with first detectable Elli-positive cells after 5 min (Fig. 6a, gating is shown in Additional file 1: Figure S4). Homing with the GASNGINAYLC peptide resulted in slightly faster uptake kinetics compared to SLWERLAYGIC peptide.

The intracellular trafficking studies revealed significant co-localization between hNET and both hNET-homing nanovehicles after 5 min incubation (Fig. 6b), when most of the FRT nanovehicles were localized inside early endosomes (Fig. 6c). The hNET-nanovehicle co-localization was higher in the case of homing with GASNGINAYLC peptide. Taken together, these results show that hNET-homing FRT nanovehicles are rapidly associating with hNET and are internalized together via endocytosis.

### Nanovehicles decorated with hNET-homing peptides show pronounced cytotoxicity for neuroblastoma cells

**Fig. 7:**
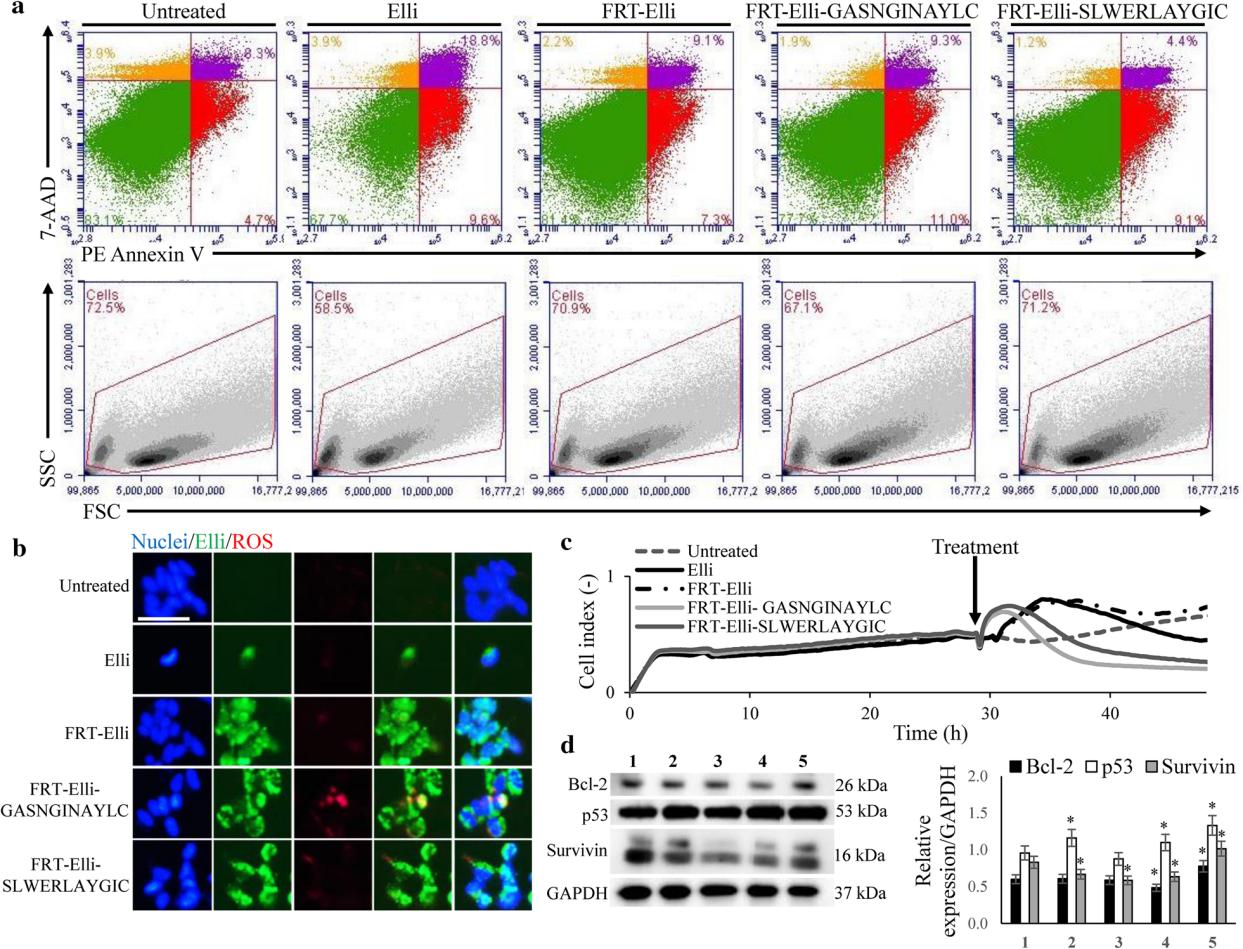
hNET-homing peptides-targeted nanovehicles show pronounced cytotoxicity, enabled by formation of ROS and deregulation of anti- and pro-apoptotic genes. **a** Induction of early and late apoptosis stained by Annexin V and 7-AAD after a 6-h treatment with 20 µM Elli in the form of free Elli, FRT-Elli, FRT-Elli-GASNGINAYLC and FRT-Elli-SLWERLAYGIC. **b** Formation of reactive oxygen species (ROS, red) after 6-h with 20 µM Elli (green) in the form of free Elli, FRT-Elli, FRT-Elli-GASNGINAYLC and FRT-Elli-SLWERLAYGIC. Nuclear counterstain (blue) was done using Hoechst 33342. Scale bar, 50 µm. **c** Real-time proliferation measurement of SH-SY5Y cells after treatment with 20 µM Elli in the form of free Elli, FRT-Elli, FRT-Elli-GASNGINAYLC and FRT-Elli-SLWERLAYGIC over the course of 24 h. Arrow shows the time-point of treatment. **d** The expression of cancer progression proteins Bcl-2, p53 and survivin upon 24 h treatment with 10 µM Elli in the form of free Elli (2), FRT-Elli (3), FRT-Elli-GASNGINAYLC (4) and FRT-Elli-SLWERLAYGIC (5) compared with expression in untreated cells (1). *indicates significance at *p *< 0.05 in relative expression between treated and untreated cells

Dual staining with PE-Annexin V and 7-AAD revealed that due to the rapid endocytosis hNET-homing nanovehicles induced significantly higher level of early apoptosis after 6 h than naked FRT-Elli nanovehicles (11% and 7.3%, respectively, Fig. 7a). Furthermore, nanovehicles utilizing homing with the GASNGINAYLC peptide led to significantly higher degree of late apoptosis compared to homing with the SLWERLAYGIC peptide (9.3% and 4.4%, respectively) and formation of significantly higher amount of reactive oxygen species (ROS) was observed with these nanovehicles compared to nanovehicles utilizing homing with SLWERLAYGIC peptide (Fig. 7b).

Moreover, real-time cell proliferation and adhesion measurements over the course of 24 h revealed a rapid influence of the hNET-homing nanovehicles on the cell adhesion and proliferation. This effect was even more pronounced than the influence of free Elli, whereas naked FRT nanovehicles supported the adhesion and proliferation of neuroblastoma cells (Fig. 7c).

Distinct deregulation in expression of proteins that are involved in cancer progression was observed after 24 h treatment with hNET-homing nanovehicles (Fig. 7d). Although free Elli treatment led to significant increase in the p53 expression while reducing the expression of survivin, Elli encapsulation into naked FRT nanovehicles resulted only in down-regulation of survivin. On the other hand, hNET-homing with GASNGINAYLC led to significant decrease in both Bcl-2 and survivin, while increasing p53. Treatment with FRT-Elli-SLWERLAYGIC resulted in increased expression of Bcl-2, p53 and survivin. Taken together, the in vitro cytotoxic tests demonstrate a better hNET-homing capability of GASNGINAYLC compared to SLWERLAYGIC.

### hNET-homing FRT-based nanovehicles are highly biocompatible

**Fig. 8:**
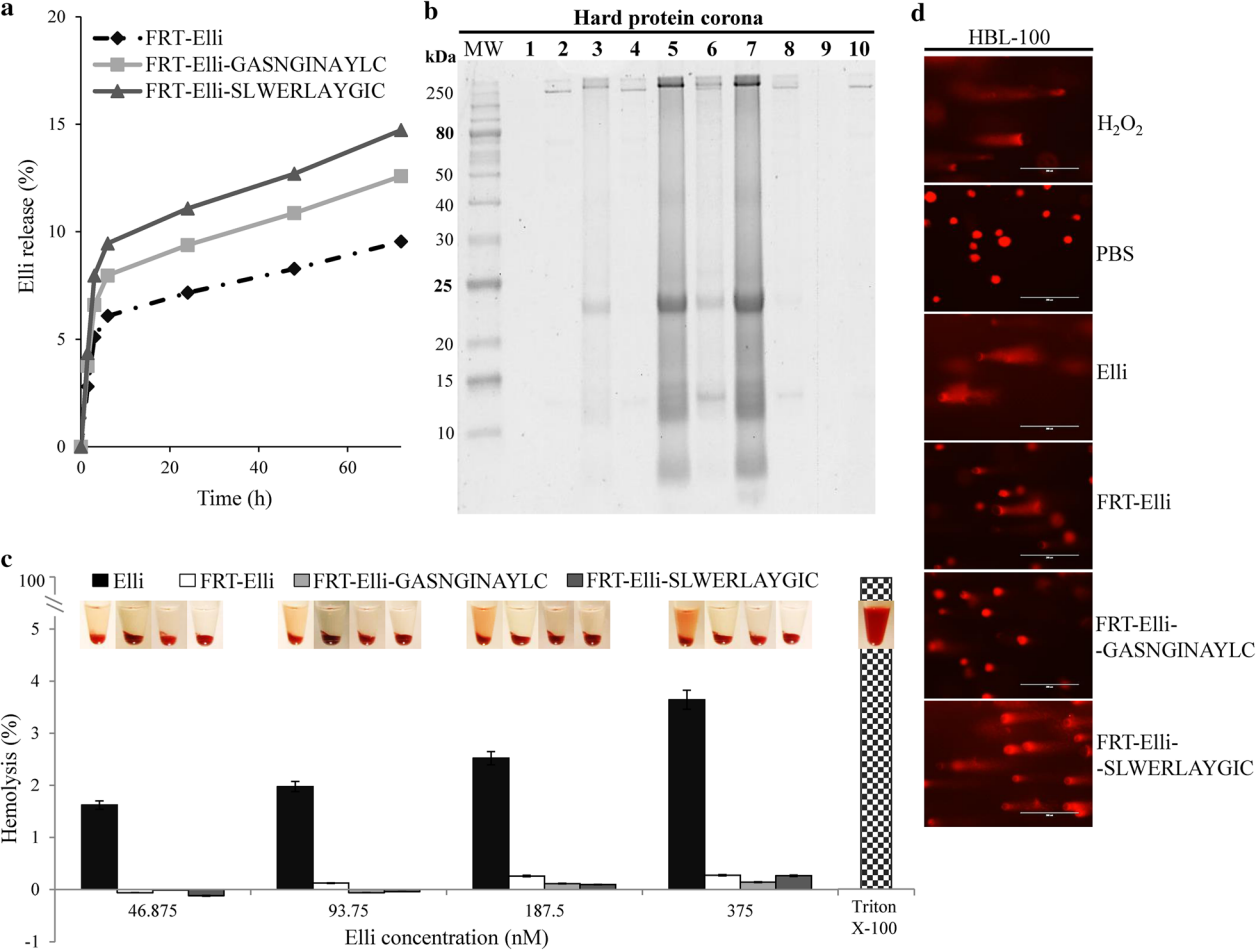
hNET-homing nanovehicles are highly biocompatible. **a** Premature release kinetics of Elli from FRT nanovehicles over 72 h in Ringer’s solution. **b** Polyacrylamide gel showing hard protein corona formation on FRT nanovehicles in fetal bovine serum (FBS). 10-250 kDa protein ladder (L), Elli without (1) and with formed hard corona (2), FRT-Elli without (3) and with formed hard corona (4), FRT-Elli-GASNGINAYLC without (5) and with formed hard corona (6), FRT-Elli-SLWERLAYGIC without (7) and with formed hard corona (8), FRT without (9) and with formed hard corona (10). **c** Hemolytic properties of free Elli, FRT-Elli, FRT-Elli-GASNGINAYLC and FRT-Elli-SLWERLAYGIC. **d** Nuclear DNA fragmentation analyzed by single-cell gel electrophoresis in off-target HBL-100 cells treated for 6 h with of 20 µM Elli in the form of free Elli, FRT-Elli, FRT-Elli-GASNGINAYLC and FRT-Elli-SLWERLAYGIC. Phosphate-buffered saline (PBS) and H_2_O_2_ were employed as negative and positive control, respectively

Encapsulation of Elli into FRT nanovehicles led to very slow plasma release kinetics (< 10% after 72 h, Fig. 8a). Although the subsequent modification with hNET-homing peptides increased this premature release, the level of released drug was still very low (< 13 and < 15% after 72 h for FRT-Elli-GASNGINAYLC and FRT-Elli-SLWERLAYGIC, respectively).

The investigation of hard protein corona formation revealed that both naked and hNET-homing nanovehicles retained their biological identity in the presence of proteins from serum with no detectable adsorbed proteins (Fig. 8b). The encapsulation of Elli and subsequent introduction of hNET homing also led to elimination of Elli intrinsic hemolytic properties (Fig. 8c). On the other hand, Elli encapsulated in the naked nanovehicles or with hNET-homing using the SLWERLAYGIC peptide still retained its genotoxicity for normal cells. Only the homing using the GASNGINAYLC peptide led to decrease of the genotoxicity (Fig. 8d). Taken together, the hNET-homing via the GASNGINAYLC peptide leads to creation of very biocompatible nanovehicles.

## Discussion

By far, hNET is the most commonly targeted membrane protein in neuroblastoma diagnosis and radiotherapy, encompassing nearly 90% expression in neuroblastoma cells [11]. Among cell surface proteins, hNET is one of the best candidates for high-resolution homology modeling due to the recent elucidation of homolog proteins dDAT and hSERT [18]. Nanomedicine-based approaches are under development for targeting hNET via antibodies [22] or non-radioactive MIBG-linked ligands [23], and provide rational less-invasive alternative strategy to radiotherapy. The present work explores a better method of targeting hNET via non-toxic homing peptides. Peptides are more customizable, feasible, and easier to synthesize and characterize than antibodies and ligands. The homing peptides GASNGINAYLC and SLWERLAYGIC were derived from the hNET sequence directly (specifically from residues 286–295/extracellular and 583–592/cytoplasmic, respectively), thus they should be highly biocompatible from immunological point of view. Solvated molecular Haddock docking showed both homing peptides to be binding in the same site inside hNET channel. In both peptides, most of the contribution to the binding was attributed to the electrostatic energy of Asn and Arg (< − 228 kJ/mol). Furthermore, the *N*-terminus displayed direct interaction with D473 in hNET channel. GASNGINAYL peptide displayed interactions between residues N7^peptide^ and K541/T544^hNET^. SLWERLAYGI peptide displayed interactions between R5^peptide^ and D473/T544^hNET^, as well as between R5^peptide^ and D473/T474^hNET^. The fact that most of these residues in hNET are not conserved in hDAT and hSERT is very promising for selectivity and will possibly decrease adverse effects of targeted payload [24]. Namely, the residues D473-T474/K541/T544 in hNET are replaced with D-H/R/H in hDAT and E-E/P/R in hSERT [25]. Structure-structure alignment of four docked peptides in hNET revealed seven overlapping residues between both peptides. This unexpected result gives insight to the possible mode of binding for both homing peptides where the site of interaction can be further explored. The negatively charged hNET channel is highly attractive to positively charged compounds such as catecholamines (e.g. norepinephrine and dopamine) [26] and χ-MrlA inhibitor peptides [27]. The peptide alignment can be used as a roadmap for in silico lead-optimization via truncation (*N*-terminus of GASNGINAYL and *C*-terminus of SLWERLAYGI), alanine scan, mutants of Asn and Arg, extension of *N*-terminus of SLWERLAYGI, etc. [28].

A secondary binding mode was found for SLWERLAYGI peptide. This binding pose puts the peptide in a position more distant from the original binding site (~ 11.5 Å between Docking D^1st^ and D^2nd^ peptide centroids) with direct contact of R5^peptide^ with Asn89^hNET^ at the opposite side of the channel. We believe this weaker binding mode can hypothetically explain the decreased efficiency later seen for this homing peptide.

MD simulations of the homing peptides, modified with and without *C*-terminus amidation and addition of cysteines, did not show large changes in secondary structures. The spectrometric methods showed that the cysteination did not affect the amide I vibrations in both FT-IR and Raman spectra. However, while cysteination contributed to helicity, it caused a 50% drop in β-sheet/backbone aggregation in the GASNGINAYL peptide as visible in the CD spectra.

We did not find acute cytotoxicity of these hNET-homing peptides; however, their distinct influence on deregulation of expression of a number of genes was observed. We found that most genes affected by the hNET-homing peptides are involved in stimulation of metabolic process, response to stress and nervous system development. Interestingly, nervous system development pathways were identified in both tested peptides, which supports hNET’s role in neuroblastoma growth and progression. Noteworthy, we identified up-regulation of *ACTR2* (ARP2 Actin Related Protein 2 Homolog) gene, which plays a role in neuritogenesis of neuronal cells, growth cone motility and filopodia formation [29]. In addition, *MAPK14* (Mitogen-activated protein kinase 14), which promotes differentiation of neuroblastoma cells into neurons mediated by adrenergic receptors and MAPK pathway [30], was also up-regulated upon exposure to both hNET-homing peptides. From the spectrum of down-regulated genes, we should highlight *ERBB3* (V-erb-b2 erythroblastic leukemia viral oncogene homolog 3). ERBB receptor tyrosine kinases (such as EGFR) influence adhesion, migration, survival, and differentiation in many types of cells and play critical roles in many malignancies [31]. Therefore, their down-regulation could be an advantageous feature associated with hNET-homing peptides-actively targeted nanomedicine. We have also revealed that hNET-homing peptides inhibited the expression of *ST7* (Suppressor of tumorigenicity 7 protein), which negatively regulates apoptotic process through regulation of genes maintaining the cellular architecture [32]. Taken together, cDNA microarray survey revealed that despite the fact the hNET-homing peptides did not exhibit direct acute cytotoxicity, their binding to hNET could affect a number of down-stream pathways towards enhancement of therapeutic efficiency.

Since the in silico hNET model used for peptide docking was constructed from a homology model of *Drosophila melanogaster*’s dopamine receptor [18], concerns about the selectivity bias of the peptides towards hDAT or hSERT might be in place. However, overexpression of hNET in comparison to the other monoamine transporters in the neuroblastoma cells was confirmed in our study.

FRT cages isolated from horse spleen have already been proven numerous times as highly biocompatible nanovehicles for targeted delivery of cytotoxic compounds into cells of various malignancies, using either small molecules, peptides or antibodies. Peptide-enabled targeted delivery of potent cytotoxic molecule Elli was performed in this study. To achieve the site-specific binding of the hNET-homing peptides onto FRT surface, similar approach as in our previous work was used [33]. Considering the results of physico-chemical characterization (morphology, size, polydispersity index, stability), it can be concluded that the hNET-homing nanovehicles are highly suitable for use in nanomedicine, as they can benefit from the effect of enhanced permeability and retention to accumulate in the mass of solid tumors [34].

Biological identity of the nanoparticles can change upon introduction to a complex environment, hampering the association of the nanovehicles with their target receptors [35]. However, the hNET-homing nanovehicles produced in this work retained their ability to rapidly associate with the target cells in presence of serum within minutes due to a negligible level of formed biomolecular corona on these nanovehicles.

Overcoming the cell plasma membrane in an efficient way is another one of the main challenges of nanomedicine nowadays [36]. To achieve the release of the active compound from FRT, cellular uptake via endocytosis is required. Namely, low endosomal pH leads to a disassembly of FRT quaternary structure and release of the physically entrapped drug molecules, which can then enter cytoplasm through diffusion [37]. Therefore, unlike other nanovehicles [36], the direct entry of FRT cages into cytoplasm is not required. hNET endocytosis has been observed in the past in conjunction with recycling of monoamines [25]. To elucidate the mechanism of entry, we performed co-localization studies between early endosomal marker Rab5 [38], hNET and the FRT nanovehicles. These proved that the hNET-homing nanovehicles readily bind to hNET and induce its endocytosis, with higher co-localization observed in the case of homing with GASNGINAYLC peptide.

The cytotoxic payload compound used in this study was Elli. Elli is known to induce apoptosis in neuroblastoma cells through formation of covalent DNA adducts [39], as well as the induction of oxidative stress [40]. We proved that the encapsulation of Elli into naked FRT nanovehicles decreased apoptosis in neuroblastoma but the apoptosis was increased after introduction of hNET-homing through the peptide GASNGINAYLC. The increased formation of ROS is probably one of the reasons for this markedly increased toxicity for neuroblastoma cells.

Furthermore, the influence of the treatment on expression of certain cancer progression proteins was observed. The neuroblastoma cells reacted to nanovehicles with hNET-homing via SLWERLAYGIC peptide by up-regulation of both pro- and anti-apoptotic proteins, namely Bcl-2, survivin and p53. The stimulation of p53, as one of the key regulators of cell cycle, can induce apoptosis. However, the concurrent Bcl-2 and survivin stimulation suggests at a functional protective mechanisms in the cells [41]. On the other hand, homing via GASNGINAYLC peptide led to up-regulation of p53 together with down-regulation of both Bcl-2 and survivin. These findings help elucidate the reason behind distinct induction of apoptosis between the two hNET-homing nanovehicles.

## Conclusions

The neuroblastoma surface protein hNET is a candidate target for nanotherapy as an alternative strategy to radiotherapy. In a proof of concept, we designed two homing peptides derived from hNET and explored their structures and interactions with hNET for nanotherapy applications; computationally and physically. GASNGINAYLC peptide was proved more efficient in drug delivery. Future work will be focused on providing better understanding of the mechanism of nanovehicle binding to hNET leading to endocytosis. Furthermore, the theoretical mode of interaction between the peptides and hNET provides insights to future homing peptide development via lead optimization and functional analysis.

## Methods

### Chemicals

The chemicals used in the study were purchased from Sigma-Aldrich (St. Louis, MO, USA) in ACS purity. Exceptions are mentioned accordingly. pH measurements were concluded with the pH electrode MICRO P (XS Instruments, Carpi, Italy). FRT-nanovehicles were formed using horse spleen FRT (cat. no. A3641) and Elli (cat. no. 324688). All antibodies were diluted in 1 mg/mL bovine serum albumin (BSA) in PBS (denoted as antibody buffer).

### High-resolution homology modeling

Two alternative strategies were used to construct two hNET homology models based on the same alignment and using the same template structures acquired from the Protein Data Bank. Modeller 9.17 [16] and SwissModel server [17] were used to construct structures hNET-M and hNET-S, respectively. Briefly, Modeller’s alignment algorithm, which takes into consideration structural positions for embedded versus solvated residues, was used to generate the alignment between canonical isoform of hNET (P23975) and dDAT crystal structure (PDB ID 4XPA), with a minor adjustment. The alignment was used to build models using both SwissModel and Modeller. All 10 models built using SwissModel were identical so only one was chosen for further evaluation and refinement. Out of 1000 models built using Modeller, the model with best molpdf score was chosen for further analysis. The numeration of amino acids was corrected using UCSF Chimera 1.10.2. [42]. MolProbity [19] was used to evaluate the models throughout the whole refinement process. According to the previous guidelines [18], the models were superposed with dDAT (PDB ID 4XPA) and hSERT (PDB ID 5I6Z) using WinCoot 0.8.3 [43] to manually fix the backbone and side-chain outliers. A final minimization (100 steps of steepest descent) was used to remove clashes. Quality-guided manual refinement by superposition was used to correct the homology models without adding a substantial number of physical errors.

### Molecular docking

Initially, about 26 α-helix fragments from hNET sequence were built via Pepfold [44] and screened by molecular docking (GRAMM-X) [45] on a low-resolution, i.e. non-refined, 3-D model of hNET constructed by SwissModel [17]. The best binding candidates with the best interaction energy *in vacuo* were GASNGINAYL (− 106.2 kJ/mol) and SLWERLAYGI (− 128.6 kJ/mol) peptides (data not shown). These two were further studied as homing peptides for hNET in more detail.

Homing peptide 3-D structures were constructed using Pepfold [44], and then minimized for docking on high-resolution hNET homology models in two stages. The first stage to identify binding site on hNET was through ClustPro 2.0 server, [20] which involves 70,000 orientations of hNET to choose the best 1000 docking models and group them into few clusters. The docking models that showed binding of peptides in the channel entrance of hNET were studied to identify a direct contact that can be used in detailed solvated docking. In the second stage, peptides were docked on hNET-M and hNET-S using Haddock 2.2 server [21]. Out of 1000 rigid-body refined models, 200 flexible models were used for docking. The 200 water-solvated dockings were grouped into clusters. The *N*-terminus of GASNGINAYL peptide was docked against D473 residue in hNET, while the *N*-terminus of SLWERLAYGI peptide was docked against E382 residue in hNET. Structure-structure multiple alignment of four docked peptides in hNET (Haddock Docking A, B, C, D^1st^ in Fig. 1e) was performed in UCSF Chimera 1.10.2. [42], using the MatchMaker module (Needleman-Wunsch algorithm, BLOSUM-62 matrix with gap opening/extension penalty 12/1).

### Molecular dynamics

Peptide structures (.pdb file format) were constructed as standard α-helix using UCSF Chimera 1.10.2. [42]. This preliminary secondary structure was chosen to avoid errors in the highly sensitive protonation processing, and also to use it as a reference in RMSD representations. Sufficient times were given later at the equilibration and production phases to allow the peptides to freely deviate from α-helix reference. The amidation of the *C*-terminus was performed by editing the file manually: (1) OXT atom was replaced with N, (2) the residue number was increased by 1, (3) the residue name was replaced with NHE, (4) amide residue was moved to the end and atom numbers were corrected. Eight peptides (with/without *C*-terminal amidation and with/without *C*-terminal Cys), were processed using H++ server [46]. Protonation in the ff14SB force field was performed at physiological pH (7.4), with default dielectric constants and salinity (0.15 mM). Topology and coordinate files for implicit water model were used for MD simulation in Amber 14 using Sander module [47].

An updated version of the Generalized Born model [48] was used for calculations of implicit water. Minimization by steepest-descent was carried out by 2500 steps. Equilibration was performed at 25 °C for 0.5 ns in steps of 1 fs. MD production was done in 50 ns intervals 8 times in steps of 2 fs, using a non-bonded cutoff of 16 Å. Trajectory analysis was done using Amber cpptraj module and R language (Bio3D) [49]. Secondary structure assignments were evaluated via DSSP method [50]. All simulations were performed on a node of 10 CPUs and 8 Gb RAM memory. Each CPU was 2× 6-core Xeon E5645 (2.40 GHz). Message passing interface was used for parallel computing.

### Peptide syntheses

GASNGINAYL, GASNGINAYLC, SLWERLAYGI and SLWERLAYGIC peptides were synthesized by Fmoc (9*H*-fluoren-9-yl methoxy carbonyl-) solid-phase synthesis on Liberty Blue Peptide Synthesizer (CEM, Matthews, NC, USA).

### Fourier transform-infrared spectroscopy

FT-IR spectra were collected on attenuated total reflection FT-IR spectrometer Invenio (Bruker). Lyophilized peptides were analyzed on a diamond crystal. FT-IR Spectrum was combined from 128 interferograms at 25 °C with 2 cm^−1^ resolution from 4000 to 650 cm^−1^.

### Circular dichroism

CD spectra were collected using Jasco model J-1500 spectropolarimeter at 298 K using quartz cells. Peptide solutions, in ACS water, were analyzed at 1 and 0.5 mM concentrations. The spectral resolution was 0.1 nm, whereas the wavelength ranges were 190–300 and 200–300 nm for path lengths of 0.1 and 1.0 mm, respectively. The analysis of secondary structure was done visually in MS Excel and via regression analysis according to Raussens et al. [51]. Briefly, the spectra were normalized to 207 nm and ellipticity at 193, 196 and 211 nm was used to calculate predictive models.

### Raman spectroscopy

Raman spectroscopy was carried out using InVia Reflex Raman microspectrometer (Renishaw, Wotton-under-Edge, UK) with excitation wavelength of 785 nm via diode laser at 150 mW power. Spectra were recorded in the range 200–2000 cm^−1^ and 2000–3000 cm^−1^. Scans of 10 s were accumulated ten times. The obtained spectra were baseline-corrected (cubic spline interpolation, selection of explicit X positions), smoothed (Savitzky-Golay, polynomial order 3, smooth window 15) and normalized in WiRE 3.4 (Renishaw).

### Tissue cultures

SH-SY5Y were purchased from Health Protection Agency Culture Collections (Salisbury, UK), UKF-NB-4 were kindly provided by prof. Tomas Eckschlager from University Hospital Motol, Prague, Czechia. Normal epithelial cell line from breast HBL-100 was purchased from American Type Culture Collection (Manassas, VA, USA). Neuroblastoma cells were cultivated in Iscove’s Modified Dulbecco’s Medium and normal breast cells were cultured in Dulbecco’s Modified Eagle’s Medium. Penicillin (100 U/mL), streptomycin (0.1 mg/mL) and FBS (10% *v/v*) were supplemented to all media, and the cells were cultured at 37 °C and 85–95% incubator (Galaxy 170 R, Eppendorf, Hamburg, Germany) with 5% CO_2_. To increase hNET expression, cells were treated with 10 µM SAHA for 24 h prior to further experiments.

### Cell viability

The cell viability was tested for cells treated for 24 h with up to 62.5 µM GASNGINAYL, GASNGINAYLC, SLWERLAYGI and SLWERLAYGIC peptides by measuring mitochondrial activity according to Tesarova et al. [52].

### RNA isolation

Total RNA was isolated using RNeasy Mini Kit (Qiagen, Hilden, Germany) from subconfluent neuroblastoma cell lines. The RNA concentration and purity was assessed with Infinite 200 PRO NanoQuant instrument (Tecan, Zürich, Switzerland) and RNA integrity was checked on Bleach gel [53].

### Electrochemical microarray

To understand the molecular response to exposure to the hNET-homing peptides, ~ 1×10^6^ of SH-SY5Y cells were treated with 50 µM GASNGINAYLC or SLWERLAYGIC (24 h). Upon isolation of RNA, microarray transcriptomic profiling was done according to Rodrigo et al. [54].

### Quantitative real-time PCR

Total RNA (2 μg) with anchored Oligo(dT)_18_ primers was used for cDNA synthesis performed by Transcriptor First Strand cDNA Synthesis Kit (Roche, Basel, Switzerland). Quantification of gene expression was performed by Luna^®^ Universal qPCR Master Mix (NEB, Beverly, MA, USA) and Mastercycler^®^ ep Realplex real-time PCR instrument (Eppendorf). Each 20 μL qPCR mix contained 100 ng cDNA and set of primers with 250 nM final concentration. The primers displayed at Additional file 1: Table S6 were designed to span exon–exon junctions by PrimerQuest Tool (IDT, IA, USA). The qPCR program was performed as follows: 5 min denaturation (95 °C), followed by 40 cycles of 20 s denaturation (95 °C) and 30 s extension (60 °C). High-resolution melting curve analysis was carried out to validate the amplification specificity. Furthermore, gel electrophoresis (ethidium bromide-stained 2% agarose gel), followed by visualization on Azure C600s imaging system (Azure Biosystems, Dublin, CA, USA) were employed to analyze the amplification specificity and control of primer-dimer formation. Threshold cycle (C_T_) was analyzed by noise band with automatic baseline drift correction using Realplex software (Eppendorf). The relative expression levels of monoamine transporters were normalized to *HPRT1* reference gene and expressed as mean ± SD ΔC_T_ values (C_T HPRT1_ − C_T SLC6A2/3/4_) from three biological replicates. Fold change differences between relative *SLCA6A2* expression and *SLCA6A3/4* expression was determined as 2^(ΔCT SLCA6A2^^−^^ΔCT SLC6A3/4)^.

### Assembly of FRT-based nanovehicles

The encapsulation of Elli into FRT was performed using a similar approach as in case of doxorubicin encapsulation shown in our previous study [33] and has already been thoroughly characterized and published elsewhere [39, 55]. Contrary to doxorubicin, Elli was dissolved in acidified water (85:1 Milli-Q water:1 M HCl) at 1 mg/mL. 200 µL of acidified Elli was mixed with 20 µL of FRT (50 mg/mL) and 100 µL of Milli-Q water, followed by mixing for 15 min, during which the FRT structure disassembled due to pH ~ 3.7. Addition of 300 µL of 200 mM Na_2_HPO_4_–NaH_2_PO_4_ buffer (pH 7.0) led to neutralization of the pH, reassembly of FRT structure and physical entrapment of Elli inside FRT cavity (creating FRT-Elli). Decoration with AuNPs, binding with hNET-homing peptides, as well as the removal of free Elli and peptides was done as in our previous paper [33]. For this purpose, the peptides were dissolved in Milli-Q and sonicated. This led to creation of FRT-Elli-GASNGINAYLC or FRT-Elli-SLWERLAYGIC, respectively.

### Physico-chemical characterization of the nanovehicles

Encapsulation efficiency of Elli was calculated based on absorbance at 420 nm. Visualization of nanovehicle morphology, together with the average size and ζ-potential measurements performed in Ringer’s solution (6.5 g/L NaCl, 0.42 g/L KCl, 0.25 g/L CaCl_2_ and 0.2 g/L of NaHCO_3_, pH 7.4) were performed as in our previous work [37]. To evaluate the colloidal stability of the nanovehicles, they were dissolved in Ringer’s solution, kept at 20 °C in a stationary rack and photodocumentation of sedimentation and/or precipitation was done for 24 h.

### Expression of L-FRT surface receptor SCARA5 and hNET

Expression of SCARA5 in ~ 1×10^6^ subconfluent cells was evaluated by western blotting. For this purpose, anti-SCARA5 (ab118894, Abcam, Cambridge, UK, 1:1000) and anti-GAPDH G-9 (sc-365062, Santa Cruz Biotechnology, Dallas, TX, USA, 1:700) antibodies were used, with overnight incubation at 4 °C. HRP-labeled anti-rabbit antibody (SAB3700831, Sigma-Aldrich) or anti-mouse antibody (P0260, Dako, Carpinteria, CA, USA), both 1:5000, together with Clarity Western ECL Blotting Substrate (Bio-Rad, Hercules, CA, USA) and Azure c600 imager (Azure Biosystems), were used. The densitometric analyses were performed using open source software Fiji ImageJ (National Institute of Health, Bethesda, MD, USA). Immunocytochemistry was carried out to evaluate the hNET expression after 24 h incubation with 0 or 10 µM SAHA. For this purpose, mouse anti-hNET antibody (MAB5620, Merck Millipore, Burlington, MA, USA, 1:500) and anti-mouse antibody labeled with Cruz Fluor™ 647 (sc362287, Santa Cruz Biotechnology, 1:500) were used. The protocol was adapted from our previous work [52].

### Uptake kinetics of the nanovehicles

A suspension of 3 × 10^5^ SH-SY5Y cells was seeded overnight into a 12-well plate. Then, cells were introduced to 20 µM Elli in the form of free Elli, FRT-Elli, FRT-Elli-GASNGINAYLC or FRT-Elli-SLWERLAYGIC for up to 24 h. At certain time points (5, 15, 30 min, 1, 2, 4, 8, 12 and 24 h), the cells were collected using accutase. Then, the cells were sedimented at 400×*g* and 4 °C for 5 min, washed once with PBS and resuspended in 3% FBS in PBS. The uptake was quantified via fluorescence using a 488-nm laser and 533/30 filter on flow cytometer BD Accuri C6 Plus (BD Biosciences, Franklin Lakes, NJ, USA). The flow rate was 35 µL/min and a minimum of 1 × 10^4^ cells was analyzed in each group.

### Nanovehicle intracellular trafficking

After formation of hNET-homing nanovehicles, FRT was labelled using cyanine 3 NHS ester (Lumiprobe, Cockeysville, MD, USA). A suspension of 5 × 10^3^ SH-SY5Y cells was seeded onto a coverslip overnight. Then, the cells were incubated with 100 µL of medium containing hNET-homing nanovehicles with 80 µM Elli for 5 min. Next, the cells were fixed using 4% formaldehyde/PBS for 10 min, followed by 3× washing with PBS. To stain intracellular hNET and Rab5, we permeabilized the cells using 0.1% Triton X-100/PBS for 5 min. After 3× washing with PBS, the plate was blocked for 1 h at 4 °C with 3% BSA/PBS. Then, the cells were incubated in 100 µL of anti-hNET (MAB5620, Merck Millipore, 1:500) or anti-Rab5 (PA3-915, Thermo Fisher Scientific, 1:250) antibodies for 16 h at 4 °C. Unbound antibodies were removed by 3× washing with PBS-T. Next, the cells were incubated for 1 h at 4 °C with anti-mouse (sc-362287, Santa Cruz Biotechnology, 1:500, for hNET) or anti-rabbit (ab150079, Abcam, 1:500, for Rab5) antibodies labelled with CruzFluor^®^ 647 or AlexaFluor^®^ 647, respectively, followed by 3× washing with PBS-T. Hoechst 33342 (1:2000 in PBS) was added for 4 min at 25 °C to counterstain nuclei. After final washing with PBS, the coverslips were mounted with SlowFade™ Diamond Antifade Mountant (Invitrogen, Carlsbad, CA, USA). Confocal laser scanning microscopy was performed with the emitted light from Cy3-labelled FRT collected within a detection window 570-624 upon irradiation by a solid state 561 nm laser. Emitted light from Elli was collected within a detection window 500–550 nm upon irradiation by a solid state 488 nm laser. Hoechst 33342 and AlexaFluor^®^ 647/CruzFluor^®^ 647 were excited with the 405 nm and 633 nm and their emissions were recorded at 460–480 nm and 645–690 nm. Micrographs were acquired using LSM 880 (Carl Zeiss, Jena, Germany). Confocal data files were processed using Zen 2.3 (blue edition, Carl Zeiss). Quantitative determination of spatial receptor-nanovehicle correlations was performed using Fiji ImageJ (National Institute of Health).

### Evaluation of induction of apoptosis

6 × 10^5^ cells were seeded overnight into a 6-well plate. After removal of the medium and washing with PBS, 1 mL of fresh medium with 20 µM Elli, either free or encapsulated in naked or hNET-homing nanovehicles was added to the cells. After 6 h, medium containing detached cells was collected and mixed with cells detached by accutase. Dual staining of translocated phosphatidylserine and cell permeability was done using the PE Annexin V Apoptosis Detection Kit I (BD Biosciences). Early/late apoptosis analyses were performed on flow cytometer BD Accuri C6 Plus (BD Biosciences). The flow rate was 35 µL/min and a minimum of 1 × 10^5^ cells was analyzed in each group. Representative plots of three independent measurements are shown.

### Analysis of formation of reactive oxygen species

To observe ROS formation in response to Elli uptake, a suspension of 1 × 10^3^ cells was seeded overnight into a 24-well plate. Cells were administered with 20 µM Elli, either free or encapsulated in naked or hNET-homing nanovehicles. After 6 h, cells were stained with CellROX^®^ Deep Red (Thermo Fisher Scientific, 1:500 in PBS). Hoechst 33342 (Invitrogen, 1:5000) was used to counterstain nuclei and the intensity of CellROX^®^ Deep Red fluorescence was checked under fluorescence microscope EVOS FL Auto Cell Imaging System (Thermo Fisher Scientific).

### Real-time cell proliferation and adhesion analysis

Cell proliferation and adhesion analysis was performed on xCELLigence RTCA DP instrument (Roche). The background impedance signal was measured using 50 µL of fresh medium. Then, 100 µL of medium containing 5 × 10^3^ cells was seeded for 28 h. Next, 200 µL of fresh medium containing 20 µM Elli, either free or encapsulated in naked or hNET-homing nanovehicles, was added. Cell proliferation and adhesion was observed every 15 min for further 24 h.

### Deregulation of expression of proteins involved in cancer progression

~ 1 × 10^6^ cells were seeded into a T-75 flask overnight. The cells were administered with 1 mL of fresh medium containing 10 µM Elli, either free or encapsulated in naked or hNET-homing nanovehicles. After 24 h, cells were lysed with RIPA buffer. 10 µg of the total proteins were loaded in loading buffer (50 mM Tris/HCl, 2% SDS and 20% glycerol), separated and electrotransferred onto a PVDF membrane. After blocking of nonspecific binding with 10% dried skimmed milk, anti-Bcl-2 (sc-7382, Santa Cruz Biotechnology, 1:200), anti-p53 DO-1 (sc-126, Santa Cruz Biotechnology, 1:250), anti-survivin (ab76424, Abcam, 1:5000) and anti-GAPDH G-9 (sc-365062, Santa Cruz Biotechnology, 1:700) antibodies were incubated with the membranes for 16 h at 4 °C. After washing, HRP-labeled anti-rabbit antibody (SAB3700831, Sigma-Aldrich) for survivin or anti-mouse antibody (P0260, Dako) for Bcl-2, p53 and GAPDH, both 1:5000, were used. Chemiluminiscent substrate Clarity Western ECL Blotting Substrate (Bio-Rad) was used, followed by detection on Azure c600 imager (Azure Biosystems). The densitometric analyses were performed using Fiji ImageJ (National Institute of Health).

### Nanovehicle biocompatibility

Premature release of Elli from the naked and hNET-homing nanovehicles was studied after their dispersion in Ringer’s solution. The samples were kept for up to 72 h at 37 °C, with periodic collection of the Ringer’s solution containing free Elli molecules and measurement of Elli content by fluorescence. To evaluate the formation of hard protein corona upon entering plasma, 50% plasma content in blood was mimicked by mixing of FBS and Elli, FRT, FRT-Elli, FRT-Elli-GASNGINAYLC and FRT-Elli-SLWERLAYGIC (200 µg/mL) in 1:1 ratio (*v/v*). To mimic other in vivo conditions, 37 °C and 600 rpm was employed for 35 min. The hard protein coronas were recovered and visualized as described in Tesarova et al. [52]. Hemotoxicity for red blood cells, as well as genotoxicity (single-cell gel electrophoresis) for off-target cells (HBL-100) were checked also according Tesarova et al. [52].

### Descriptive statistics and data processing

Mean ± SD were used to express the obtained results. Software GraphPad (GraphPad Software, San Diego, CA, USA) was employed for analyses. We used unpaired Student’s t-test to statistically evaluate the differences between two groups or one-way ANOVA, followed by unpaired Student’s t-test, to compare multiple groups. Microsoft Office PowerPoint software (Redmond, WA, USA) or Fiji Image J (National Institute of Health) were employed for processing of schematics and figures, unless otherwise mentioned. The transcriptomic data were analyzed using DAVID Functional Annotation Bioinformatics Microarray Analysis software (v6.8, https://david.ncifcrf.gov/home.jsp, Frederick National Laboratory for Cancer Research, Frederick, MD, USA).

## Supplementary information

**Additional file 1: Figure S1.** Alignment of hNET sequence (P23975) and dDAT crystal structure (PDB ID: 4XPA) using Modeller. **Table S1.** Summary of manual refinement and quality control of homology models hNET-M and hNET-S. **Table S2.** Summary of ClustPro geometric docking. **Table S3.** Summary of Haddock solvated docking. **Figure S2.** Generalized Born implicit-water molecular dynamics simulations (400 ns) at physiological pH 7.4 of homing peptides variants GASNGINAYL(Cys/-NH_2_) (**A**, **a-c**) and SLWERLAYGIC(Cys/-NH_2_) (**B**, **a-c**). Left panels show time evolution of peptide secondary structures and Root-mean square-deviations (RMSD, Å), whereas right panels show percentage of secondary structure distribution among residues and average in the last column. **Table S4.** Full list of genes deregulated in SH-SY5Y cells after incubation with GASNGINAYLC peptide. The list contains genes, which were up- or down-regulated in three independent analyzes (*n* = 3). Shown are only the genes with fold ratio > 1.5 or < 0.5. **Table S5.** Full list of genes deregulated in SH-SY5Y cells after incubation with SLWERLAYGIC peptide. The list contains genes, which were up- or down-regulated in three independent analyzes (*n* = 3). Shown are only the genes with fold ratio > 1.5 or < 0.5. **Figure S3.** hNET (red) immunofluorescence in wild type neuroblastoma cells and after 24-h treatment with SAHA, cell autofluorescence and fluorescence after incubation with anti-mouse fluorescent secondary antibody. Hoechst 33342 (blue) was used to counterstain nuclei. Scale bar, 50 µm. **Figure S4.** Scatter plots showing the gating of cells on forward *vs.* side scatter for the measurement of uptake kinetics of hNET-homing nanovehicles encapsulating Elli. **Table S6.** List of primers employed for qPCR analysis of *SLC6A2*, *SLC6A3* and *SLC6A4* expression.

## Data Availability

The datasets supporting the conclusions of this article are included within the article (and its additional files).

## Abbreviations

Asn: Asparagine
Arg: Arginine
AuNPs: Gold nanoparticles
BSA: Bovine serum albumin
CD: Circular dichroism
Cys: Cysteine
Elli: Ellipticine
FBS: Fetal bovine serum
FRT: Ferritin
FT-IR: Fourier transform-infrared spectroscopy
hDAT: Human dopamine transporter
hNET: Human norepinephrine transporter
hSERT: Human serotonin transporter
MD: Molecular dynamics
MIBG: Metaiodobenzylguanidine
PBS: Phosphate-buffered saline
PNTs: Peripheral neuroblastic tumors
qPCR: Quantitative polymerase chain reaction
RMSD: Root-mean square-deviation
ROS: Reactive oxygen species
SAHA: Suberanilohydroxamic acid
SCARA5: Scavenger receptor class A member 5
SCGE: Single-cell gel electrophoresis
SD: Standard deviation
Trp: Tryptophan
