# Supplementary material for: Norepinephrine transporter-derived homing peptides enable rapid endocytosis of drug delivery nanovehicles into neuroblastoma cells
Source: J Nanobiotechnology. 2020 Jul 13;18:95. doi: 10.1186/s12951-020-00654-x (PMC7359476; doi:10.1186/s12951-020-00654-x)
Supplement: Supplementary file 1 — Additional file 1: Figure S1. Alignment of hNET sequence (P23975) and dDAT crystal structure (PDB ID: 4XPA) using Modeller. Table S1. Summary of manual refinement and quality control of homology models hNET-M and hNET-S. Table S2. Summary of ClustPro geometric docking. Table S3. Summary of Haddock solvated docking. Figure S2. Generalized Born implicit-water molecular dynamics simulations (400 ns) at physiological pH 7.4 of homing peptides variants GASNGINAYL(Cys/-NH2) (A, a-c) and SLWERLAYGIC(Cys/-NH2) (B, a-c). Left panels show time evolution of peptide secondary structures and Root-mean square-deviations (RMSD, Å), whereas right panels show percentage of secondary structure distribution among residues and average in the last column. Table S4. Full list of genes deregulated in SH-SY5Y cells after incubation with GASNGINAYLC peptide. The list contains genes, which were up- or down-regulated in three independent analyzes (n = 3). Shown are only the genes with fold ratio > 1.5 or < 0.5. Table S5. Full list of genes deregulated in SH-SY5Y cells after incubation with SLWERLAYGIC peptide. The list contains genes, which were up- or down-regulated in three independent analyzes (n = 3). Shown are only the genes with fold ratio > 1.5 or < 0.5. Figure S3. hNET (red) immunofluorescence in wild type neuroblastoma cells and after 24-h treatment with SAHA, cell autofluorescence and fluorescence after incubation with anti-mouse fluorescent secondary antibody. Hoechst 33342 (blue) was used to counterstain nuclei. Scale bar, 50 µm. Figure S4. Scatter plots showing the gating of cells on forward vs. side scatter for the measurement of uptake kinetics of hNET-homing nanovehicles encapsulating Elli. Table S6. List of primers employed for qPCR analysis of SLC6A2, SLC6A3 and SLC6A4 expression. [file 12951_2020_654_MOESM1_ESM.docx]

**Norepinephrine Transporter-Derived Homing Peptides Enable Rapid Endocytosis of Drug Delivery Nanovehicles into Neuroblastoma Cells**

Yazan Haddad^1,2^, Marketa Charousova^1,2^, Hana Zivotska^1,2^, Zbynek Splichal^1,2^, Miguel Angel Merlos Rodrigo^1,2^, Hana Michalkova^1,2^, Sona Krizkova^1,2^, Barbora Tesarova^1,2^, Petr Vitek^3^, Kamila Stokowa-Soltys^4^, David Hynek^1,2^, Lukas Richtera^1,2^, Vedran Milosavljevic^1,2^, Simona Dostalova^1,2*^, Zbynek Heger ^1,2*^

**Figure S1.** Alignment of hNET sequence (P23975) and dDAT crystal structure (PDB ID: 4XPA) using Modeller.

**Table S1.** Summary of manual refinement and quality control of homology models hNET-M and hNET-S.

|  | **hNET-M model** | | **hNET-S model** | |
| --- | --- | --- | --- | --- |
| **Refinement** |  | |  | |
| Phi/Psi changes | K201, L584 | | G217, V532, L584 | |
| Rotamers superposed to dDAT | 163_mt_, L146_tt_, P188_cg exo_, K201_tptp_, E212_mt-10_, V282_t_, T283_p_, K303_mttt_, K334_mttt_, I349_mt_, L398_mt_, T450_m_, L455_tp_, E488_mt-10_, L515_mt_, L530_tp_, P542_cg exo_, K576_tttp_, L584_pp_, L588_tp_, P594_cg exo_ | | D75_p-10_, L100_mt_, F101_m-85_, L146_tp_, L169_tt_, N170_p30_, N192_m120_, N292_t-20_, D378_m-20_, L398_pp_, D418_p-10_, V498_m_, D499_p-10_, N503_t-20_, Q507_tt_, V524_t_, V532_m_, V533_p_, F540_t80_, K576_tttp_ | |
| Rotamers superposed to hSERT | K61_tttt_, T99_p_, I103_mt_, C131_m_, L163_tp_, S165_m_, Y214_t80_, V218_p_, Q236_mp0_, L237_mt_, V256_m_, V276_t_, V279_t_, L302_tp_, E304_mm-40_, I315_tp_, F329_m-30_, C351_m_, V356_m_, I389_m_, E393_tt_, V449_p_, V468_t_, R518_ttp85_, Y572_t80_, I592_pp_ | | T99_p_, I130_mm_, Y161_t80_, Y162_m-85_, S165_m_, Y214_t80_, V218_p_, Q236_mp0_, M242_ttt_, V247_p_, T268_p_, L302_tp_, E304_mm-40_, I309_mt_, I315_tp_, V356_m_, S357_m_, E393_tt_, V449_p_, R518_ttp85_, Y572_t80_ | |
| **Quality** | **Before** | **After** | **Before** | **After** |
| Clashscore | 87.7 (0^th^ percentile) | 27.18 (18^th^ percentile) | 4.51 (95^th^ percentile) | 7.56 (84^th^ percentile) |
| Poor rotamers | 6 (1.31%) | 2 (0.48%) | 5 (1.09%) | 3 (0.71%) |
| Ramachandran outliers | 2 (0.37%) | 0 (0.00%) | 1 (0.18%) | 0 (0.00%) |
| *C*-beta outliers | 4 (0.8%) | 7 (1.41%) | 3 (0.6%) | 9 (1.81%) |
| MolProbity score | 2.54 (45^th^ percentile) | 2.15 (67^th^ percentile) | 1.42 (97^th^ percentile) | 1.6 (92^nd^ percentile) |

**Table S2.** Summary of ClustPro geometric docking.

| **ClustPro Docking** | **A** | **B** | **C** | **D** |
| --- | --- | --- | --- | --- |
| Ligand | GASNGINAYL | GASNGINAYL | SLWERLAYGI | SLWERLAYGI |
| Receptor | hNET-M | hNET-S | hNET-M | hNET-S |
| Clusters | 9 | 8 | 9 | 9 |
| Order among clusters | 2^nd^ | 2^nd^ | 1^st^ | 2^nd^ |
| Models in chosen cluster | 236/1000 | 213/1000 | 427/1000 | 266/1000 |
| Median score | -733.9 | -735.0 | -881.7 | -803.8 |

**Table S3.** Summary of Haddock solvated docking.

| **Haddock Solvated docking** | **A** | **B** | **C** | **D** | |
| --- | --- | --- | --- | --- | --- |
| Ligand | GASNGINAYL | GASNGINAYL | SLWERLAYGI | SLWERLAYGI | |
| Receptor | hNET-M | hNET-S | hNET-M | hNET-S | |
| Interacting residue in ligand | G1 | G1 | S1 | S1 | |
| Interacting residue in receptor | D473 | D473 | E382 | E382 | |
| Total number of Clusters | 2 | 7 | 1 | 2 | |
| Order among Clusters | 1^st^ | 1^st^ | 1^st^ | 1^st^ | 2^nd^ |
| Haddock score | -95.9 ± 6.3 | -72.0 ± 3.3 | -84.9 ± 2.9 | -80.1 ± 6.7 | -25.6 ± 6.0 |
| Cluster size/total | 190/198 | 15/184 | 200/200 | 194/199 | 5/199 |
| RMSD from the overall lowest-energy structure | 0.4 ± 0.2 | 0.4 ± 0.2 | 0.4 ± 0.2 | 0.4 ± 0.2 | 1.7 ± 0.1 |
| Van der Waals energy | -31.4 ± 2.9 | -34.9 ± 6.8 | -35.5 ± 3.7 | 41.6 ± 3.8 | -19.4 ± 1.1 |
| Electrostatic energy | -292.3 ± 23.2 | -253.3 ± 39.9 | -228.9 ± 39.5 | -260.5 ± 22.0 | -82.9 ± 19.9 |
| Desolvation energy | -6.1 ± 9.2 | 13.6 ± 6.0 | -3.6 ± 3.2 | 13.6 ± 4.6 | 10.4 ± 8.0 |
| Restraints violation energy | 0.0 ± 0.0 | 0.0 ± 0.0 | 0.0 ± 0.0 | 0.0 ± 0.0 | 0.0 ± 0.0 |
| Buried surface area | 1162.8 ± 61.1 | 1198.5 ± 106.7 | 1184 ± 6.4 | 1402.8 ± 60.6 | 746.5 ± 122.8 |
| Z-score | -1.0 | -1.8 | 0.0 | -1.0 | 1.0 |


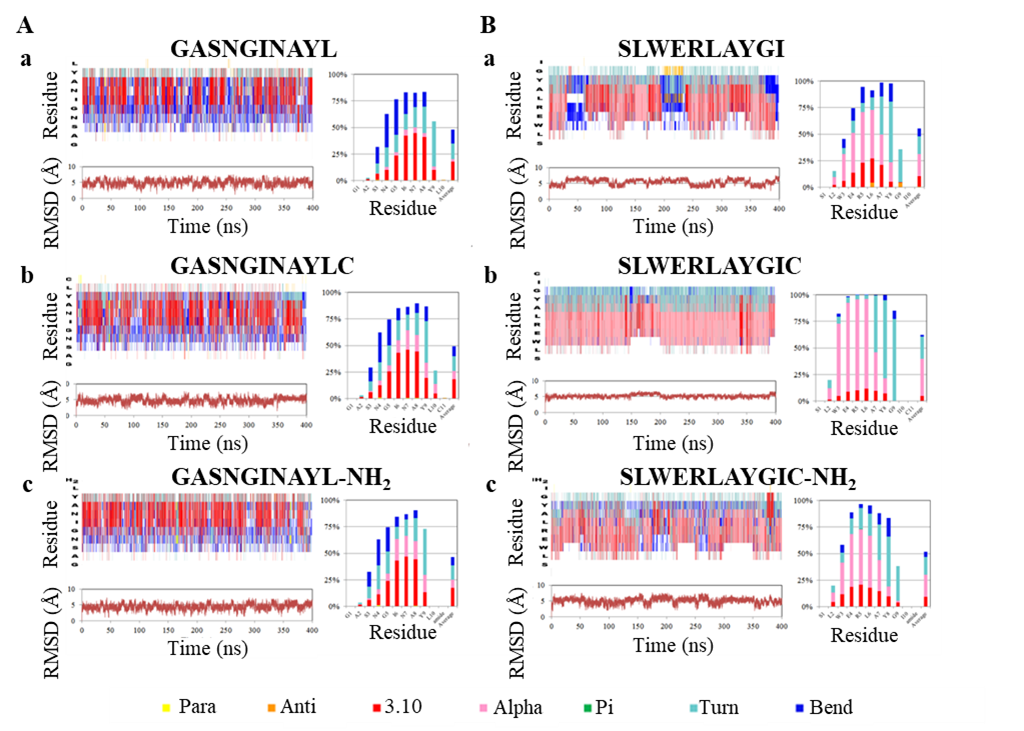


**Figure S2.** Generalized Born implicit-water molecular dynamics simulations (400 ns) at physiological pH 7.4 of homing peptides variants GASNGINAYL(Cys/-NH_2_) (**A**, **a-c**) and SLWERLAYGIC(Cys/-NH_2_) (**B**, **a-c**). Left panels show time evolution of peptide secondary structures and Root-mean square-deviations (RMSD, Å), whereas right panels show percentage of secondary structure distribution among residues and average in the last column.

**Table S4.** Full list of genes deregulated in SH-SY5Y cells after incubation with GASNGINAYLC peptide. The list contains genes, which were up- or down-regulated in three independent analyzes (*n* = 3). Shown are only the genes with fold ratio >1.5 or <0.5.

| **Up-regulation** | | | | |
| --- | --- | --- | --- | --- |
| **Gene** | **Symbol** | **NCBI database** | **FR*** | **SD (n = 3)** |
| Reticulocalbin 1 | RCN1 | NM_002901 | 18.15 | 3.451 |
| ARP2 actin-related protein 2 homolog | ACTR2 | NM_001005386 | 2.53 | 0.201 |
| Scavenger receptor cysteine-rich type 1 protein M160 | M160 | NM_174941 | 2.51 | 0.075 |
| KH-type splicing regulatory protein | KHSRP | NM_003685 | 2.25 | 0.497 |
| Mitogen-activated protein kinase 14 | MAPK14 | NM_139013 | 2.22 | 0.026 |
| RAD21 homolog | RAD21 | NM_006265 | 1.98 | 0.018 |
| Adipose differentiation-related protein | ADFP | NM_001122 | 1.82 | 0.064 |
| Thymidine kinase 1 | TK1 | NM_003258 | 1.78 | 0.220 |
| S-adenosylhomocysteine hydrolase | AHCY | NM_000687 | 1.68 | 0.288 |
| Dystonin | DST | NM_183380 | 1.66 | 0.045 |
| SRY (sex determining region Y)-box 4 | SOX4 | NM_003107 | 1.61 | 0.229 |
| Solute carrier family 25, member 6 | SLC25A4 | NM_001151 | 1.61 | 0.426 |
| Creatine kinase brain | CKB | NM_001823 | 1.61 | 0.004 |
| Protein kinase C | PRKCQ | NM_006257 | 1.54 | 0.137 |
| Choline kinase alpha | CHKA | NM_001277 | 1.52 | 0.147 |
| **Down-regulation** | | | | |
| **Gene** | **Symbol** | **NCBI database** | **FR*** | **SD (n = 3)** |
| Hepsin (transmembrane protease, serine 1) | HPN | NM_002151 | 0.56 | 0.018 |
| Proline 4-hydroxylase | P4HB | NM_000918 | 0.55 | 0.064 |
| Zinc finger protein 36, C3H type, homolog | ZFP36 | NM_003407 | 0.54 | 0.220 |
| V-erb-b2 erythroblastic leukemia viral oncogene homolog 3 | ERBB3 | NM_001005915 | 0.50 | 0.288 |
| Fc fragment of IgG, low affinity IIIa, receptor (CD16a) | FCGR3A | NM_000570 | 0.42 | 0.045 |
| Thymidylate synthetase | TYMS | NM_001071 | 0.41 | 0.029 |
| Ubiquitin-conjugating enzyme E2C | UBE2C | NM_181800 | 0.23 | 0.041 |
| Retinol binding protein 4, plasma | RBP4 | NM_006744 | 0.11 | 0.198 |
| ST7 overlapping transcript 2 | ST7 | NM_021908 | 0.09 | 0.419 |

*FR, fold ratio.

**Table S5.** Full list of genes deregulated in SH-SY5Y cells after incubation with SLWERLAYGIC peptide. The list contains genes, which were up- or down-regulated in three independent analyzes (*n* = 3). Shown are only the genes with fold ratio >1.5 or <0.5.

| **Up-regulation** | | | | |
| --- | --- | --- | --- | --- |
| **Gene** | **Symbol** | **NCBI database** | **FR*** | **SD (n = 3)** |
| ARP2 actin-related protein 2 homolog | ACTR2 | NM_001005386 | 8.23 | 1.224 |
| Reticulocalbin 1 | RCN1 | NM_002901 | 3.10 | 0.411 |
| Mitogen-activated protein kinase 14 | MAPK14 | NM_139013 | 2.73 | 0.064 |
| Choline phosphotransferase 1 | CHPT1 | NM_020244 | 1.96 | 0.029 |
| KH-type splicing regulatory protein | KHSRP | NM_003685 | 2.25 | 0.497 |
| FXYD domain containing ion transport regulator 5 | FXYD5 | NM_014164 | 1.76 | 0.132 |
| Melanoma cell adhesion molecule | MCAM | NM_006500 | 1.74 | 0.091 |
| Trefoil factor 3 | TFF3 | NM_003226 | 1.72 | 0.147 |
| Alpha-2-macroglobulin | A2M | NM_000014 | 1.71 | 0.236 |
| Thymidine kinase 1 | TK1 | NM_003258 | 1.62 | 0.066 |
| Creatine kinase brain | CKB | NM_001823 | 1.54 | 0.198 |
| Dystonin | DST | NM_183380 | 1.53 | 0.044 |
| LUC7-like | LUC7L | NM_018032 | 1.52 | 0.011 |
| F-box protein 31 | FBXO31 | NM_024735 | 1.51 | 0.411 |
| S-adenosylhomocysteine hydrolase | AHCY | NM_000687 | 1.50 | 0.206 |
| **Down-regulation** | | | | |
| **Gene** | **Symbol** | **NCBI database** | **FR*** | **SD (n = 3)** |
| V-erb-b2 erythroblastic leukemia viral oncogene homolog 3 | ERBB3 | NM_001005915 | 0.59 | 0.061 |
| Paternally expressed 3 | PEG3 |  | 0.58 | 0.018 |
| Stanniocalcin 1 | STC1 |  | 0.58 | 0.155 |
| Mitogen-activated protein kinase 12 | MAPK12 |  | 0.55 | 0.220 |
| Spondin 2, extracellular matrix protein | SPON2 |  | 0.52 | 0.068 |
| MLF1 interacting protein | MLF1IP |  | 0.51 | 0.470 |
| Dynactin | DCTN4 |  | 0.44 | 0.081 |
| ST7 overlapping transcript 2 | ST7 |  | 0.14 | 0.110 |

*FR, fold ratio.


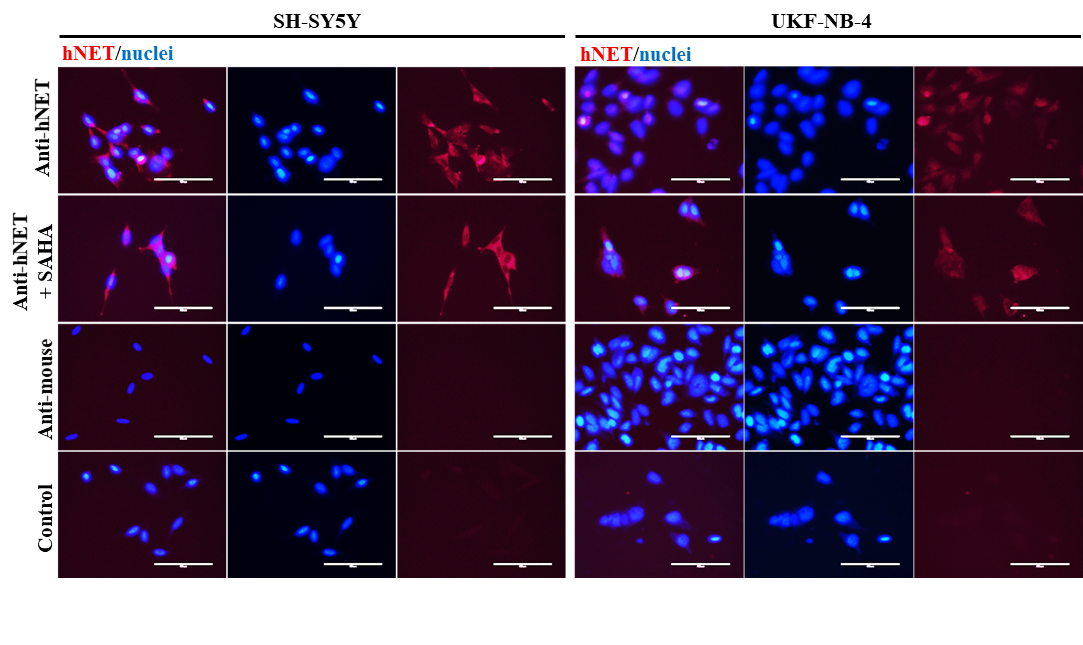


**Figure S3.** hNET (red) immunofluorescence in wild type neuroblastoma cells and after 24-h treatment with SAHA, cell autofluorescence and fluorescence after incubation with anti-mouse fluorescent secondary antibody. Hoechst 33342 (blue) was used to counterstain nuclei. Scale bar 50 µm.

**
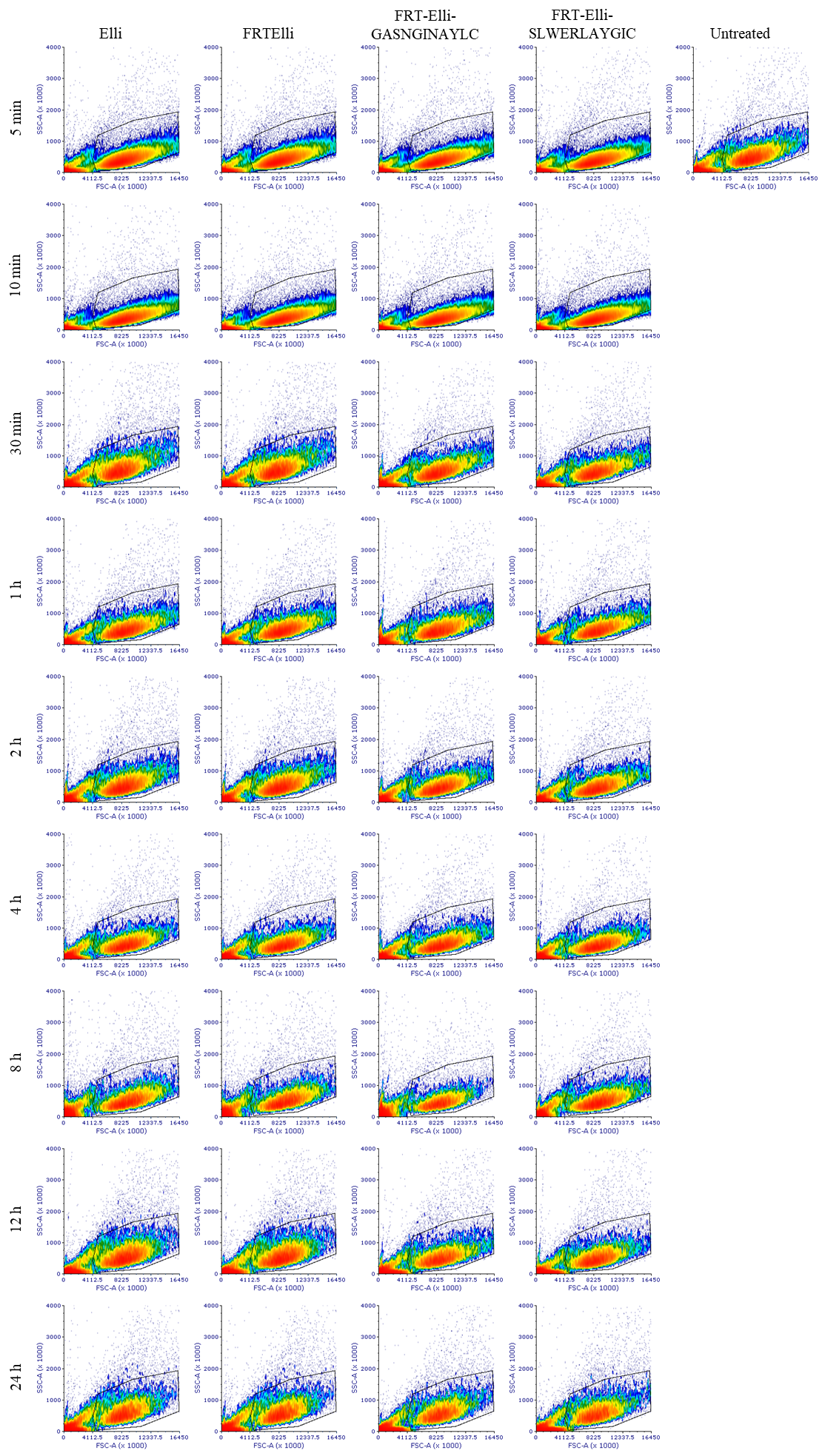
**

**Figure S4.** Scatter plots showing the gating of cells on forward vs. side scatter for the measurement of uptake kinetics of hNET-homing nanovehicles encapsulating Elli.

**Table S6.** List of primers employed for qPCR analysis of *SLC6A2*, *SLC6A3* and *SLC6A4* expression.

| **Full name/Function** | **Gene** |  | **Primer (5´-3´)** | **Product size**  **(nt)** |
| --- | --- | --- | --- | --- |
| Solute carrier family 6 member 2 | *SLC6A2* | F | CTGTATCACCAGCTTCGTCTC | 111 |
| *Norepinephrine transporter* |  | R | ACTAGGCCAGCTCCTTCT |  |
| Solute carrier family 6 member 3 | *SLC6A3* | F | TCAGGGAAGGTGGTATGGAT | 108 |
| *Dopamine transporter* |  | R | GTATGCTCTGATGCCGTCTATG |  |
| Solute carrier family 6 member 4 | *SLC6A4* | F | CCCTCTGTTTCTCCTGTTCATC | 114 |
| *Serotonine transporter* |  | R | GTTCCTATGCAGTAACCCAAGA |  |
| Hypoxanthine phosphoribosyltransferase 1 | *HPRT1* | F | TGGCGTCGTGATTAGTGATG | 128 |
| *Generation of purine nucleotides (reference gene)* |  | R | GACGTTCAGTCCTGTCCATAAT |  |
